# Mechanochemically-derived iron atoms on defective boron nitride for stable propylene production†

**DOI:** 10.1039/d4ey00123k

**Published:** 2024-08-07

**Authors:** Gian Marco Beshara, Ivan Surin, Mikhail Agrachev, Henrik Eliasson, Tatiana Otroshchenko, Frank Krumeich, Rolf Erni, Evgenii V. Kondratenko, Javier Pérez-Ramírez

**Affiliations:** a Institute for Chemical and Bioengineering, Department of Chemistry and Applied Biosciences, ETH Zurich Vladimir-Prelog-Weg 1 8093 Zurich Switzerland jpr@chem.ethz.ch; b Laboratory of Physical Chemistry, Department of Chemistry and Applied Biosciences, ETH Zurich Vladimir-Prelog-Weg 2 8093 Zurich Switzerland; c Electron Microscopy Center, Empa – Swiss Federal Laboratories for Materials Science and Technology (EMPA) Uberlandstrasse 129 8600 Dubendorf Switzerland; d Advanced Methods for Applied Catalysis, Leibniz-Institut fur Katalyse Albert Einstein-Strasse 29a 18059 Rostock Germany; e Laboratory of Inorganic Chemistry, Department of Chemistry and Applied Biosciences, ETH Zurich Vladimir-Prelog-Weg 1 8093 Zurich Switzerland

## Abstract

Single-atom catalysts (SACs), possessing a uniform metal site structure, are a promising class of materials for selective oxidations of hydrocarbons. However, their design for targeted applications requires careful choice of metal–host combinations and suitable synthetic techniques. Here, we report iron atoms stabilised on defective hexagonal boron nitride (h-BN) *via* mechanochemical activation in a ball mill as an effective catalyst for propylene production *via* N_2_O-mediated oxidative propane dehydrogenation (N_2_O-ODHP), reaching 95% selectivity at 6% propane conversion and maintaining stable performance for 40 h on stream. This solvent-free synthesis allows simultaneous carrier exfoliation and surface defect generation, creating anchoring sites for catalytically-active iron atoms. The incorporation of a small metal quantity (0.5 wt%) predominantly generates a mix of atomically-dispersed Fe^2+^ and Fe^3+^ species, as confirmed by combining advanced microscopy and electron paramagnetic resonance, UV-vis and X-ray photoelectron spectroscopy analyses. Single-atom iron favours selective propylene formation, while metal oxide nanoparticles yield large quantities of CO_*x*_ and cracking by-products. The lack of acidic functionalities on h-BN, hindering coke formation, and firm stabilisation of Fe sites, preventing metal sintering, ensure stable operation. These findings showcase N_2_O-ODHP as a promising propylene production technology and foster wider adoption of mechanochemical activation as a viable method for SACs synthesis.

## Abstract 2

Mechanochemical activation in a ball mill effectively stabilizes Fe single atoms on a defective boron nitride host, resulting in a stable and selective catalyst for propylene production *via* N_2_O-mediated oxidative dehydrogenation of propane.

## Graphical abstract

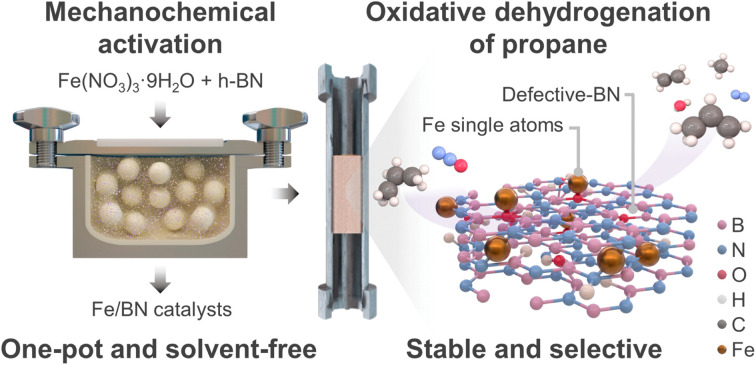

**Broader context**

Single-atom catalysts (SACs) hold the potential to significantly reduce the environmental burden of the chemical industry by unlocking selective and more efficient catalytic pathways. Identifying optimal metal–host combinations to minimise side reactions is key, but it also requires suitable and sustainable synthetic methodologies to stabilise metal centers on the carrier. In this work, we showcase mechanochemical activation in a ball mill as a one-pot, solvent-free synthesis approach for stabilising iron single atoms on defective hexagonal boron nitride and demonstrate the potential of the resultant material in N_2_O-mediated oxidative dehydrogenation of propane to propylene. This catalytic technology offers an attractive alternative to the conventional non-oxidative propane dehydrogenation, significantly lowering the operating temperature and improving propylene selectivity, owing to the uniform and isolated nature of Fe sites. Furthermore, the use of defective boron nitride, a support with negligible surface acidity, allows suppression of commonly encountered coking phenomena and hence stable operation. Finally, we encourage further studies to evaluate the viability of mechanochemically-derived h-BN-supported SACs for diverse applications.

## Introduction

Selective oxidations of hydrocarbons, where control over the degree of oxidation of the substrate is key, represent one of the biggest challenges to the modern chemical industry.^1–5^ Recent advances in the development of single-atom catalysts (SACs) offer a promising avenue for tackling it, as uniformity of metal site structure can enable improved control over the reaction selectivity and thus make processes more efficient.^6–8^ However, the design of suitable hosts with tailored surface properties and controlled reactivity under reaction conditions remains a challenge.^9,10^ Hexagonal boron nitride, h-BN, a 2D-layered material, has recently attracted attention as a catalyst support due to its high chemical and thermal stability.^11^ However, existing research is primarily focused on transition metal nanoparticle-based (NPs) systems supported on h-BN,^12–15^ while little attention is given to its potential as a host for SACs. This fact can be attributed to the difficulty in stabilising isolated metal cations on h-BN due to its low surface area and the lack of anchoring sites.^11^ Energy-intensive thermal and harsh chemical exfoliation methods have been reported to address the former,^16,17^ while the latter can be tackled through controlled introduction of structural defects, such as B- and N-vacancies.^18^ Mechanochemical treatments,^19^ in particular ball milling, can be powerful and versatile tools for addressing these challenges, having been previously recognised for their efficacy in exfoliating 2D-layered materials.^20,21^ Moreover, large mechanical forces and high local temperatures generated during the milling can result in surface restructuring^22,23^ by introducing defect sites (*e.g.*, adatoms, heteroatoms, vacancies) which can anchor metal species upon their subsequent deposition.^24^ In fact, ball milling has been proposed as a valid method for direct synthesis of SACs on other supports, such as carbons and metal oxides.^25^ Nevertheless, it has never been implemented for transition metal-BN based systems and could therefore be an attractive approach for facile and efficient preparation of stable SACs for complex chemical transformations. One such highly relevant reaction is nitrous oxide-mediated oxidative dehydrogenation of propane (N_2_O-ODHP) to propylene.^26^ It is a more selective and potentially more sustainable alternative to the non-oxidative propane dehydrogenation (PDH), as demonstrated with O_2_-ODHP and CO_2_-ODHP.^27,28^ In view of ongoing efforts to develop a technology to produce cost-effective N_2_O,^29–31^ utilizing it as selective oxidant in ODHP could be a promising strategy for meeting the ever-increasing global demand for this platform chemical. However, comprehensive techno-economic analysis (TEA) and life cycle assessment (LCA) studies are essential to fully evaluate its environmental and economic viability. Recent years have seen extensive research into the synergistic effects of Fe species in efficiently activating N_2_O, already industrially exploited for N_2_O decomposition from stationary and mobile sources.^32^ This remarkable synergy remains critically relevant for N_2_O-ODHP. There, isolated α-Fe sites within the pores of MFI zeolites,^33^ when used in tandem with the soft N_2_O oxidant instead of traditionally applied O_2_, have long been recognised to be highly efficient at catalysing propylene formation, while avoiding deep oxidation.^34,35^ However, due to the acidic nature of the zeolite, coke formation leads to pore blockage and rapid deactivation, limiting the applicability of this catalyst.^36–40^ Indeed, the lower oxidising potential of N_2_O, despite its selective nature, is insufficient for complete coke oxidation to CO_*x*_, unlike O_2_-ODHP.^26^ Therefore, dispersing Fe single atoms (SAs) on a host devoid of acidic protonic sites, *i.e.* defective h-BN, could be a viable approach for exploiting the ability of isolated Fe sites to activate N_2_O and to selectively yield propylene from propane without losing activity.

Herein, we report Fe atoms stabilised over defective h-BN as an efficient catalyst for N_2_O-ODHP. The mechanochemical synthesis *via* ball milling is revealed to play a pivotal role in generating the optimal catalyst structure, simultaneously exfoliating h-BN, increasing the available surface area, and introducing structural defects within the crystalline lattice, which serve to anchor metal species. This process primarily results in the atomic dispersion of a mixture of Fe^2+^ and Fe^3+^ species, as evidenced by electron paramagnetic, UV-vis, X-ray photoelectron spectroscopy and advanced microscopy analyses. The degree of Fe dispersion was found to have a strong effect on catalytic performance, with Fe SAs favouring selective propylene formation, while larger agglomerates and NPs favoured the formation of cracking and CO_*x*_ products. Remarkably, isolated Fe sites could maintain their speciation and showed no loss of activity for over 40 h on stream, marking this system as the first stable catalyst for N_2_O-ODHP. Our results highlight ball milling as a promising technique and defective h-BN as a practical support for SACs synthesis and demonstrate how targeted catalyst engineering could make the use of N_2_O for ODHP a compelling strategy for propylene production.

## Results and discussion

### Mechanochemical activation *via* ball milling

**Fig. 1:**
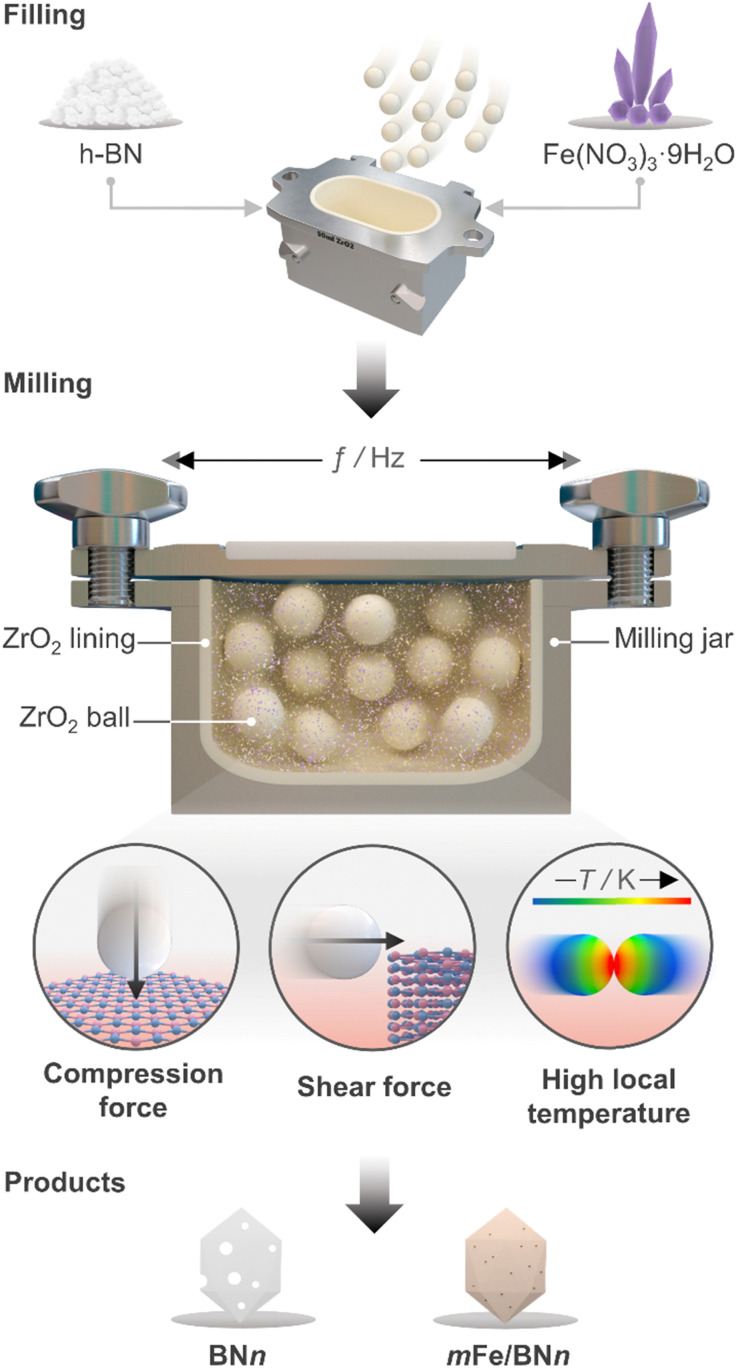
Schematic representation of the milling jar used for preparation of *m*Fe/BN*n* (*m* = metal content in wt% and *n* = milling time in h) catalysts *via* ball milling and the associated mechanophysical processes occurring during the milling process.

Ball milling was selected as a one-pot synthetic approach to attain the stabilisation of isolated Fe centres over h-BN due to its efficacy in dispersing metal species on carriers, as extensively reported for carbon- and metal oxide-supported SACs.^41^ The mechanochemical activation is driven by the kinetic energy of the grinding media.^42^ The energy is then transferred to the milled mixture, resulting in high local temperatures and plasma formation, thereby facilitating bond cleavage and surface restructuring. Moreover, by enhancing the defect density and oxygen content of the host, this approach can modulate the coordination sphere of SACs.^25^ Additionally, ball milling is a straightforward and scalable synthetic methodology, which can often be applied without the use of a solvent (*i.e.*, dry milling) and without requiring any subsequent thermal treatment of the catalytic material. Depending on the milling duration and, therefore, on the energy consumption, ball milling can be a more sustainable approach for the synthesis of SACs.^43^ As depicted in Fig. 1, pristine h-BN and Fe(NO_3_)_3_ hydrate, according to the targeted nominal metal content (0–2 wt%), were loaded in the milling jar together with the grinding balls. A selected number of cycles was applied according to the desired treatment time. While ball milling is known as one of the most effective exfoliation method for h-BN,^20–24^ the use of such a technique to directly synthesise catalysts by dispersing metal species on this carrier has never been reported in the literature, in contrast to other 2D-layered materials, such as graphene, and carbon nitride.^25^ Moreover, to directly assess the impact of the synthesis methodology on the final catalyst architecture, we employed incipient wetness impregnation (IWI), a common approach to prepare SACs,^44^ in order to generate a catalyst platform with variable Fe content, in analogy with ball milling (Table S1, ESI†). Inductively coupled plasma optical emission spectroscopy (ICP-OES) confirmed that the Fe content closely matched the nominal value for all materials (Table S1, ESI†).

**Fig. 2:**
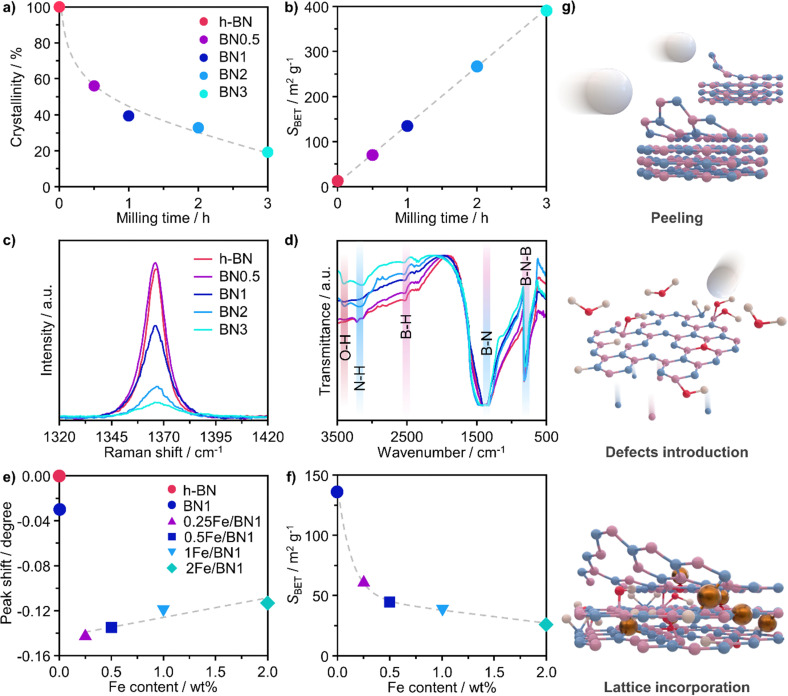
Structural properties of as-prepared milled BN and Fe/BN1 samples. (a) Relative crystallinity, determined by XRD with an internal reference, and (b) specific surface area, *S*_BET_, as a function of milling time; (c) Raman and (d) FTIR spectra of BN samples after varying milling times. (e) Shift in (002) peak position of h-BN crystalline structure, determined by XRD, and (f) *S*_BET_ as a function of Fe content. (g) Schematic representation of the dynamic changes to the catalyst architecture during the synthesis.

A crucial aspect to understand morphological properties of the host and its interactions with Fe species is the crystal structure. The lattice of h-BN is characterised by hexagonal basal sheets stacked along the *c*-direction.^11^ The AAA stacking configuration, which differs from that of graphite (ABA), provides slight ionic bonding interactions between boron and nitrogen atoms of adjacent layers. For this reason, the exfoliation of h-BN is more challenging compared to that of graphite.^45^ Furthermore, the outcome of ball milling is highly dependent on the shape and material of the milling jar, the number and size of milling balls, type of motion, frequency applied, and milling time. Now, with the aim of comprehensively understanding and rationalising the impact of treatment time on the host structure, a series of bare h-BN samples (BN*n*, *n* = milling time in h) obtained after varying milling durations was synthesised and characterised in-depth. Determination of the crystal structure of the catalysts by X-ray diffraction (XRD) showed characteristic reflections of h-BN lattice, wherein the (001) reflections represent the stacking of the BN sheets, while the (*hk*0) reflections are indicative of the order within the basal sheets. The XRD patterns of the BN*n* samples are reported in Fig. S2a (ESI†). In general, changes in the relative intensity and broadening of the peaks with increasing milling time were observed. Specifically, the (100), (101) and (102) reflections exhibited broadening without complete disappearance, indicating the notable presence of small crystalline domains, ultimately suggesting that the overall honeycomb structure is marginally preserved. These variations in the reflections may stem from the reduced particle dimensions, fluctuating strain and localised correlated disorder.^46^ Moreover, the (002) and (004) reflections broadened significantly after 3 h of milling treatment. This strongly suggests a significant decrease in the correlation length of ordered stacking in the *c*-direction.^46^ In this regard, an internal reference was adopted to directly quantify the relative degree of crystallinity of BN*n* samples compared to the pristine h-BN, thereby monitoring the peeling of the h-BN particles (Fig. 2a). As a result of the complex interaction of mechanical forces on the stacked structure of h-BN, progressive reduction in the length of ordered stacking was revealed, halving only after 0.5 h and further decreasing with longer milling time. This finding was generally in line with harsher conditions typical of dry milling compared to the solvent-assisted counterpart and points towards the capability of this technique to induce modification and damage the crystalline structure of h-BN within a limited time.^25^ To further corroborate the peeling action promoted by intense shear forces, the Brunauer–Emmett–Teller specific surface area (*S*_BET_) was determined by N_2_ sorption. The BN*n* samples showed a marked linear increase of *S*_BET_ as function of milling time (Fig. 2b). Generally, longer milling time led to a greater exposure of surfaces and edges, resulting in a significant increase of the surface area from 13 to almost 400 m^2^ g^−1^ with concomitant mesopores generation (Table S1, ESI†). This is an intriguing outcome, as previous reports have not been able to achieve similar results in terms of surface area for such short milling times.^18,23,24^ The disparity may be attributed to the optimal selection in the set of operating conditions of the treatment.

To track the generation of defects within the crystalline lattice of h-BN during the milling process, the BN*n* samples were investigated by means of Raman spectroscopy (Fig. 2c). In all samples, the Raman feature at 1365–1367 cm^−1^ could be attributed to the G-band corresponding to the E_2g_ phonon vibrational mode caused by the in-plane vibration in opposite direction of B and N atoms.^47^ The G-band consistently became weaker and broader with longer milling time. Of interest is the remarkable dependence of its full-width at half-maximum (*FWHM*) on the milling time. Prior to ball milling, pristine h-BN presented an average *FWHM* of 9.3 cm^−1^. The value gradually increased for longer milling time until doubling after only 3 h, indicating the introduction of defects within the horizontal plane, aligning with previous reports (Table S2, ESI†).^20,48^ Moreover, Raman spectroscopy is a valuable tool for layer identification of h-BN, enabling us to observe the shift of in-plane phonon modes within the range of 1365–1370 cm^−1^.^11^ Indeed, a discernible blue-shift was observed in the BN*n* samples compared to the pristine h-BN bulk peak (1365 cm^−1^). This shift became more prominent with longer milling times, as demonstrated by BN3 (1367 cm^−1^). This finding provides additional evidence for the gradual exfoliation of h-BN throughout the treatment process during which the number of stacked layers decreased due to the peeling action.

Fourier transform infrared spectroscopy (FTIR) provided additional insights into the surface structure of the materials. Spectra presented in Fig. 2d offer a detailed comparison between pristine h-BN and BN*n* samples. The IR spectrum of pristine h-BN presented lattice vibration modes predominantly associated with covalent bonds between N and B atoms. Notably, two distinct absorption bands centred at 1378 cm^−1^ and at 816 cm^−1^ are observed, which correspond to the E_1u_ in-plane B–N bond stretching vibration and A_2u_ out-of-plane B–N–B bending vibration.^49,50^ Following the milling treatment, significant modifications to the spectra of exfoliated samples were observed. The B–N in-plane vibrational mode exhibited broadening and shifted upfield by 22 cm^−1^ to 1400 cm^−1^. This indicates notable changes in lattice vibrations attributed to alterations of the crystal structure as a result of the milling process. In spite of this, the out-of-plane B–N–B bending mode remained consistent at 816 cm^−1^, indicating the retention of h-BN's characteristic honeycomb structure and therefore the overall structural integrity of the sp^2^ lattice, in line with another report.^23^ Notably, an increment in the transmittance of the B–N–B bending mode compared to the B–N in-plane vibrational mode implies cleavage of B–N–B bonds induced during ball milling, leading to lattice dislocation and disruption. A distinctive observation pre- and post-ball milling was the emergence of bands centred at 3400, 3200 and 2500 cm^−1^, attributed to O–H, N–H and B–H stretching bands, respectively.^47^ This implies the formation of hydroxyl, amino and borane groups, thus the functionalization of exfoliated h-BN. The surface heterogeneity stems from the reaction between uncoordinated B and N atoms, as a consequence of the B–N–B cleavage, and the moisture contained in the ambient air present in the milling jar.

When the Fe precursor salt was added with h-BN powder prior to the milling process, noticeable changes in the crystalline structure of the final material were observed. Fig. S2b (ESI†) shows the XRD patterns of Fe/BN1 catalyst with variable Fe content milled for 1 h. It is relevant to note that all the peaks detected were assigned to the characteristic reflections of h-BN crystalline structure. This indicates that Fe(NO_3_)_3_·9H_2_O was fully decomposed and Fe was well-dispersed as SAs or formed either amorphous or crystalline phases. For the latter case, the NPs formed could be too small or too few in number to produce sufficiently sharp reflections. Moreover, to assess the influence of the metal precursor salt used, two samples with FeCl_3_ were synthesized. The XRD pattern revealed a reflection at 2*θ* = 32.6°, suggesting the presence of FeCl_2_ and indicating incomplete decomposition of the precursor after 1 h of treatment. However, extending the synthesis duration to a total of 2 h resulted in the disappearance of this reflection in the diffractogram of 0.5Fe_Cl_/BN2, indicating that synthesis parameters must be carefully optimized to achieve full decomposition of the selected precursor salt (Fig. S3, ESI†). In general, the presence of Fe had a discernible effect on the long-range order of the resultant material when compared to the BN1. The decrease in the relative peak intensity of (001) reflections and the broadening of peaks were less prominent in the presence of Fe, resulting in a pattern closely resembling that of pristine h-BN. Notably, Fe demonstrated a tendency to maintain the crystal order characteristic of the h-BN phase for an extended milling duration. This phenomenon raised several questions, including the extent to which Fe affects the milling treatment, and the overall impact of the metal loading on the resulting architecture of the catalyst. During the milling process, some of the kinetic energy transferred from the machine to the grinding balls gets dissipated as thermal energy, resulting in high local temperatures and hence the decomposition of the Fe precursor salt. In this regard, nitrate salts are well-known for their thermal instability. It can be hypothesised that the endothermic decomposition of the salt could potentially diminish the energy available for the exfoliation of h-BN. However, this explanation alone is not sufficient to substantiate the observed phenomenon, since the energy required for the dehydration and decomposition of the nitrate salt into the corresponding oxide can be considered relatively minor compared to the energy input into the system, depending on the type of metal precursor adopted. Instead, a more comprehensive explanation was found by considering the ability of Fe to form coordination bonds with atoms in the h-BN lattice. In this regard, following the decomposition of the nitrate precursor during the exfoliation process, it is postulated that the Fe ions were incorporated into the defects created in the crystalline lattice, such as N and B vacancies, 
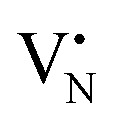
 and 
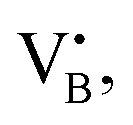
 respectively.^45^ As a result, the presence of Fe could serve not only to establish bonding interactions in crystallographic positions within a single layer. It could also actively interact with adjacent layers, intricately connecting and interlocking multiple layers of h-BN. To verify whether Fe ions were incorporated into the h-BN lattice, the increase in the interlayer distance was investigated by analysing the shifts of the main peak at 2*θ* = 26.6° of the h-BN lattice, after correcting for sample displacement with an internal standard (*i.e*, Al_2_O_3_).^51^ The shift in the peak position with respect to pristine h-BN showed a clear dependence on the metal content (Fig. 2e). For low metal content, higher shifts were obtained, indicating the expansion of the interlayer distance. Due to the difference in the atomic radii, such increased spacing may stem from the incorporation of Fe ions within defective sites as 
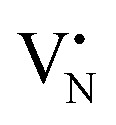
 and 
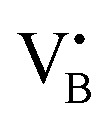
 in the h-BN structure. To explain the trend obtained, in-depth characterization of the Fe/BN1 catalysts was carried out. Raman spectroscopy revealed that for high Fe content, the intensity of the G-band closely resembled that of pristine h-BN, indicating reduced B–N bond cleavage during milling (Fig. S4, ESI†). No peaks corresponding to Fe NPs were detected with a reasonable signal-to-noise ratio. Most importantly, higher metal loadings corresponded to reduced G-band *FWHM* values, with 2Fe/BN1 exhibiting an average value of 9.7 cm^−1^, suggesting a diminished defect density with increased metal loading (Table S2, ESI†). This outcome can be explained as Fe is known as nitriding catalyst.^52^ Indeed, in a study focused on BN nanotubes synthesis from B powder and flowing gaseous NH_3_, Li *et al.* noted an increase in B–N bond formation when Fe(NO_3_)_3_ was introduced before synthesis.^53^ Thus, the catalyst structure obtained for Fe/BN1 samples emerged from the complex interplay of mechanical forces and elevated local temperatures, facilitating bond cleavage, and the capability of Fe to form covalent bonds with the support and promote new B–N bond formation. Additionally, presence of Fe hindered the peeling process, resulting in less exfoliation (Fig. 2f), which is in line with a study by Yoon *et al.* which predicted that the exfoliation process of graphene will become more energy intensive upon introduction of intercalating elements.^54^ By analogy, we can expect that at high Fe contents, instead of integration within the defects in the BN lattice, Fe species get trapped between the layers, preventing their separation.

### Architecture of Fe/BN catalyst

**Fig. 3:**
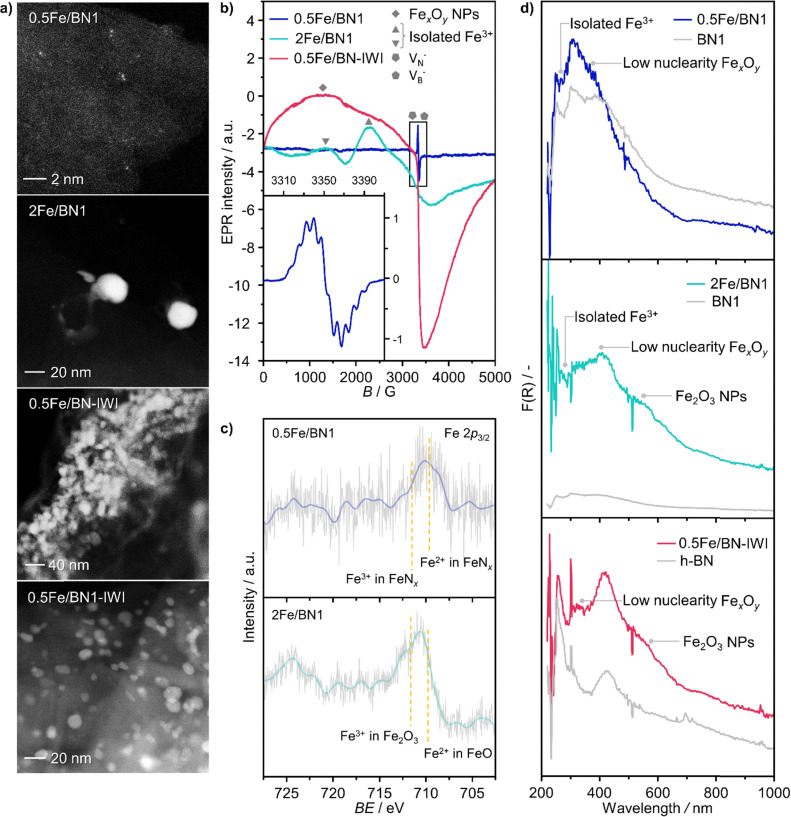
Characterization of selected as-prepared Fe/BN catalysts. (a) HAADF-STEM micrographs and (b) EPR spectra acquired at 293 K. Different symbols used for isolated paramagnetic Fe^3+^ sites correspond to species in different coordination environments. In 0.5Fe/BN1, no signal corresponding to isolated Fe^3+^ and Fe_*x*_O_*y*_ NPs is detected. The inset shows hyperfine splitting, characteristic of an electron in a nitrogen and boron vacancy (
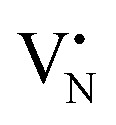
 and 
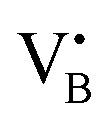
). (c) Fe 2p_3/2_ XPS spectra, demonstrating the prevalence of the low-valent Fe^2+^ species in 0.5Fe/BN1. (d) UV-vis spectra acquired at 723 K in N_2_ atmosphere, corroborating the isolated nature of iron in 0.5Fe/BN1 predominantly in a low-valent cationic state.

Following the characterization of the materials’ structural properties, degree of Fe dispersion was evaluated by means of high-angle annular dark-field scanning transmission electron microscopy (HAADF-STEM). The acquired micrographs showed that high metal content (2 wt%) resulted in a heterogeneous Fe speciation, encompassing NPs with radius ranging from 1 to 5 nm, down to sub-nanometer clusters and single atoms (Fig. 3a and Fig. S5, ESI†). This observation could be attributed to the insufficient number of defects generated during the mechanochemical activation, limiting the accessible anchoring points on the host. However, a significant improvement in terms of metal dispersion was observed when the Fe content was reduced to 1 wt%. Intriguingly, micrographs of 1Fe/BN1 revealed Fe-enriched regions as patches over the host surface as the predominant Fe species, indicating a successful reduction in the nuclearity of the metal species (Fig. S6, ESI†). These regions exhibited a distinct appearance compared to a typical nanoparticle and could be interpreted as regions with a higher concentration of low nuclearity Fe entities. In stark contrast, in 0.5Fe/BN1 Fe was mostly dispersed as SAs, indicated by the well-dispersed bright dots of minute proportions (<0.2 nm), with eventual low-nuclearity clusters present (Fig. S7, ESI†). This remarkable observation served as compelling evidence that through the facile process of ball milling, adopting an appropriate Fe content alongside a tailored treatment time to produce high surface defect density, the generation of h-BN-supported SACs was achievable. Conversely, when IWI was adopted, microscopy revealed Fe dispersed on the host in the form of NPs, regardless of metal content (Fig. 3 and Fig. S8, ESI†). Clearly, the presence of Fe NPs in all materials synthesised *via* IWI concluded this technique as unable to produce catalysts with atomically-dispersed Fe species. Likely, this limitation stems from the low surface area of commercially available h-BN (13 m^2^ g^−1^). To substantiate this assertion, we opted to impregnate the h-BN after 1-hour ball milling treatment, *i.e.* BN1, targeting a metal content equal to 0.5 wt%. This support exhibited a surface area tenfold greater compared to the pristine (136 m^2^ g^−1^, Table S1, ESI†), thereby enhancing the likelihood of obtaining isolated Fe species *via* IWI. Nevertheless, the materials obtained after annealing in air and N_2_ atmospheres, which was required to decompose the nitrate precursor, showed distinct characteristics. Annealing in air resulted in a uniform distribution of well-shaped Fe NPs on the carrier, whereas the N_2_-annealed sample exhibited higher dispersion but still did not yield Fe SAs as effectively as the 0.5Fe/BN1 sample obtained *via* mechanochemical activation. Notably, the materials post-annealing still exhibited greater surface area than 0.5Fe/BN1. This finding underscores the crucial role of mechanochemical activation, where compression and shear forces, and high local temperatures, synergistically maximised the stabilisation of Fe SAs species on the host.

To further corroborate the dispersion degree and investigate oxidation state and local coordination of Fe, continuous wave X-band EPR spectroscopy was used. The EPR spectrum of the 0.5Fe/BN1 sample measured at room temperature (Fig. 3b) consists of a narrow signal around *g* = 2.003, with a clearly resolved splitting into 10 equidistant lines. The simulation of the signal showed that it could be well-reproduced by two overlapping components (Fig. S9, ESI†). The first one consisted of a single unpaired electron (electron spin *S* = 1/2) hyperfine coupled with three equivalent B nuclei, giving rise to the observed signal splitting. Indeed, the two stable B isotopes are ^11^B (nuclear spin *I* = 3/2, natural abundance 80.42%) and ^10^B (*I* = 3, natural abundance 19.58%). A coupling of an electronic *S* = 1/2 with three ^11^B gave rise to 2·*n*(B)·*I* + 1 = 10 lines separated by the isotropic hyperfine constant (if the contribution of the anisotropic hyperfine coupling is neglected). The configurations where ^10^B was present gave rise to poorly resolved components, which mostly contribute to the inhomogeneous line broadening. This component can therefore be attributed to a 
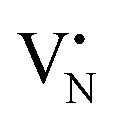
 with a single trapped electron, which is known and has been previously observed in several studies.^55–57^ The second component was a broader, poorly resolved approximately isotropic signal with *g* = 2.0036, which can be attributed to a different paramagnetic defect on the BN support, possibly a 
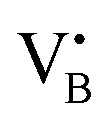
. No signals assignable to Fe single sites, particles or small aggregates were observed. At 10 K (Fig. S10, ESI†) a weak signal appeared with effective *g* = 4.3, which is typical of magnetically isolated Fe^3+^ in highly distorted orthorhombic sites, with zero field splitting (ZFS) stronger than the Zeeman interaction and the ratio of the ZFS parameters *D*/*E* = 3. Nevertheless, the intensity of this signal was within the range of the typical Fe^3+^ impurities, suggesting that Fe, whose presence was confirmed *via* ICP-OES (Table S1, ESI†), may predominantly exist in the EPR-silent divalent oxidation state. Moreover, the absence of any feature typical of Fe_*x*_O_*y*_ NPs, which exhibit characteristic ferro/antiferromagnetic or superparamagnetic signals, implies that likely Fe^2+^ was well-dispersed and most likely only present as SAs. In contrast, the room temperature spectrum of the 2Fe/BN1 sample showed a series of broad features covering the whole field range from 0 to 5000 G. The spectrum measured at 10 K showed the same signals with different relative intensities. Specifically, the signal around *g* = 4.3 appeared to be stronger. Simulations (Fig. S11, ESI†) show that the spectrum consisted of the same orthorhombic Fe^3+^ signal already observed at low temperature for 0.5Fe/BN1 (although significantly stronger and well-visible at room temperature) and an additional, less distorted Fe^3+^ site with ZFS parameters *D* = 3900 MHz and *E* = 500 MHz. The narrow distribution of ZFS parameters indicates that this Fe^3+^ site corresponded to a specific, well-defined coordination geometry. No clear evidence of ferromagnetic features due to Fe_*x*_O_*y*_ nanoparticles was observed, both at room and low temperature. However, their presence was detected *via* microscopy (Fig. 3a) and characteristic signals for this peculiar nanostructure were not visible because typically magnetically-coupled systems give very broad signals, which in this case are most likely hidden by the strong and broad paramagnetic signals due to Fe^3+^ single sites. The spectrum of the 0.5Fe/BN-IWI sample showed a very strong, extremely broad and anisotropic signal which undergoes a pronounced shift and broadening at 10 K. These characteristics and temperature dependence are typical for superparamagnetic single-domain particles and therefore the signal could be attributed to Fe_*x*_O_*y*_ NPs. This indicates a poor dispersion degree and strong aggregation of Fe in this sample. Note that the 
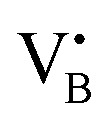
 and 
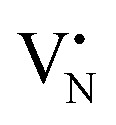
 defects signals were also observed for the 2Fe/BN1 and 0.5Fe/BN-IWI samples but were less prominent and partially hidden by single atom Fe^3+^ and Fe_*x*_O_*y*_ features.

To further assess the oxidation state of Fe in Fe/BN1 catalysts, Fe 2p_3/2_ X-ray photoelectron spectroscopic (XPS) analysis was performed. The spectrum of 0.5Fe/BN1 showed poor signal resolution due to its low metal content, yet it remained clearly discernible. Most importantly, the spectrum predominantly comprises two peaks, in good agreement with Fe^2+^ and Fe^3+^ species within FeN_*x*_ complexes, each centred at 709.60 and 711.40 eV, respectively.^58^ As suggested by EPR spectroscopy, Fe species are likely to be predominantly present in a divalent state. Conversely, in 2Fe/BN1, the Fe spectrum showed a more complex ensemble of peaks yet prominently featured a peak centred at 711.99 eV, indicative of Fe^3+^ within Fe_2_O_3_ oxide structure.^59^ The dominant contribution of this peak likely hides any minor presence of Fe^2+^ and Fe^3+^ within FeN_*x*_ complexes, as confirmed by advanced microscopy and EPR spectroscopy. Moreover, the presence of oxygen in Fe/BN1 catalysts can be considered a confirmation of the successful functionalisation of the catalyst surface (Fig. S12, ESI†). The higher signal intensity observed for 0.5Fe/BN1 compared to 2Fe/BN1 aligns with the previously discussed detrimental effect of Fe on defect generation during the mechanochemical activation.

UV-vis (200–1000 nm) absorption spectroscopy was used to further elucidate the nature of Fe species in the catalysts. In order to gain further insight into the nanostructure of the catalysts, the supports h-BN and BN1 were initially examined to be able later to detect potential shifts and alterations in the electronic structure resulting from the binding of Fe ions to the O- and N-functionalities in the support. Despite being optically transparent across the near UV-vis-NIR wavelength range,^60^ h-BN demonstrated a pronounced absorption peak in the deep UV range (200–220 nm) owing to its anisotropic structure.^61^ Moreover, it is known that the spectrum of h-BN is constituted by a first interval (200–240 nm) whose peaks origin from the contribution of the direct interband transitions across the bandgap. Consistently with the literature, three absorption peaks on the left of the intense peak have been detected as the phonon replica of the latter. For higher wavelength (240–1000 nm), the second interval consists of a defect induced broad continuum composed by many weak peaks.^62^ After exfoliation, a slight blue-shift in the absorption edge of BN1 was observed, which can be ascribed to the milling treatment, and therefore the reduction in numbers of stacked layers.^62^ Most importantly, Fe/BN catalysts exhibited heightened optical absorption. This can be attributed to the presence of additional intermediate energy states due to Fe doping and to the increased absorbance of Fe species.^63^ In the context of Fe-based catalysts, this characterization technique has been extensively employed for Fe-containing zeolites, wherein O → Fe^3+^ ligand-to-metal-charge-transfer (LMCT) bands could be observed.^64^ It is widely acknowledged that Fe^3+^ sites typically exhibit two LMCT bands associated with t1 → t2 and t1 → e transitions.^65^ The d–d transitions of Fe^3+^ ions are spin- and symmetry-forbidden,^66^ while those of Fe^2+^ ions are characterized by extremely weak transitions occurring in the NIR range, reported only at high Fe concentrations.^67^ In our UV-vis spectra, we did not observe absorptions corresponding to the d–d transitions of Fe^2+^, likely because these signals are obscured by those originating from the defective structure of h-BN. The absorption bands observed in the analysed samples are likely indicative of O → Fe^3+^ LMCT transitions. However, a contribution from O → Fe^2+^ LMCT transitions cannot be excluded. Isolated Fe^3+^ species manifest LMCT transitions in the high-energy region of the spectrum, typically below 300 nm. Note that the peaks in the regions 220–250 nm and 250–300 nm correspond to the LMCT from the nonbonding valence orbital O(2p) to the crystal field orbital Fe(3d) of isolated Fe atoms in tetrahedral (T_d_) and octahedral (O_h_) coordination, respectively.^68,69^ Considering species with higher nuclearity, the LMCT bands shift towards low energy regions. In the range 300–350 nm, several reports have documented the presence of dinuclear Fe^3+^–O–Fe^3+^ complex, while the range 350–450 nm is typical of polynuclear O_h_-Fe^3+^–oxo complexes. In the following, these species exhibiting broad bands spanning from 300 to 450 nm are referred to as low nuclearity clusters (Fe_*x*_O_*y*_).^70–72^ Bulk-like Fe_2_O_3_ NPs typically shows reflections in the region 450–800 nm, where typical absorption bands of the hematite-like structure can be observed, as 2(^6^A_1_) → 2(^4^T_1_) and ^6^A_1_ → ^4^T_2_ at 529 and 649 nm, respectively.^73^

As expected, 2Fe/BN1 spectrum showed a variety of peaks attributable to the heterogeneous Fe speciation, going from large Fe_2_O_3_ NPs down to SAs. Indeed, T_d_- and O_h_-Fe^3+^ peaks could be observed in the range 220–250 nm and 250–300 nm. According to EPR spectroscopy, the multiple peaks in these regions can be attributed to T_d_- and O_h_-Fe^3+^ with different degree of geometry distortion. The peak centered at 410 nm evidenced the presence of low nuclearity Fe_*x*_O_*y*_ species. A weak and broad peak at 529 nm, with a shoulder at 600 nm, can be attributed to the presence of Fe_2_O_3_ NPs. Note that for 0.5Fe/BN1, the same references corresponding to the aforementioned O-LMCT bands were employed, although Fe may be bound to N atoms. The spectrum of 0.5Fe/BN1 shows the presence of several absorption peaks in the range 220–250 nm and 250–300 nm corresponding to T_d_- and O_h_-Fe^3+^ species, respectively. Additionally, minor peaks in the range 300–400 nm are attributed to sporadic low nuclearity Fe_*x*_O_*y*_ clusters, also observed *via* microscopy (Fig. S7, ESI†). For wavelengths exceeding 450 nm, no distinguishable peaks compared to the background are detected, confirming the absence of Fe_2_O_3_ NPs. Moreover, the signal of 0.5Fe/BN1 exhibited a shift to lower energy compared to BN1, corroborating the integration of Fe ions into the lattice of the support.^63^ Nevertheless, the Fe signal observed in this catalyst is notably subdued in comparison to its counterpart with equivalent loading, 0.5Fe/BN-IWI. Given the high sensitivity towards trivalent Fe species of this characterization method, the low Fe signal further supports the predominance of isolated low-valent cationic Fe species in 0.5Fe/BN1, in agreement with XPS and EPR spectroscopy. The reduction of Fe^3+^ may be mediated by electron-rich species, such as 
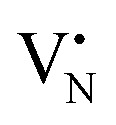
 and 
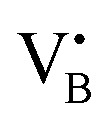
, produced after B–N–B cleavage during the milling treatment, whose presence has been demonstrated *via* EPR spectroscopy (*vide supra*). At higher Fe loadings, the extent of Fe^3+^ reduction is lower, as fewer electron-rich species are produced during mechanochemical activation, resulting in the predominant trivalent oxidation state of iron.

### Fe/BN catalysts for N_2_O-ODHP

**Fig. 4:**
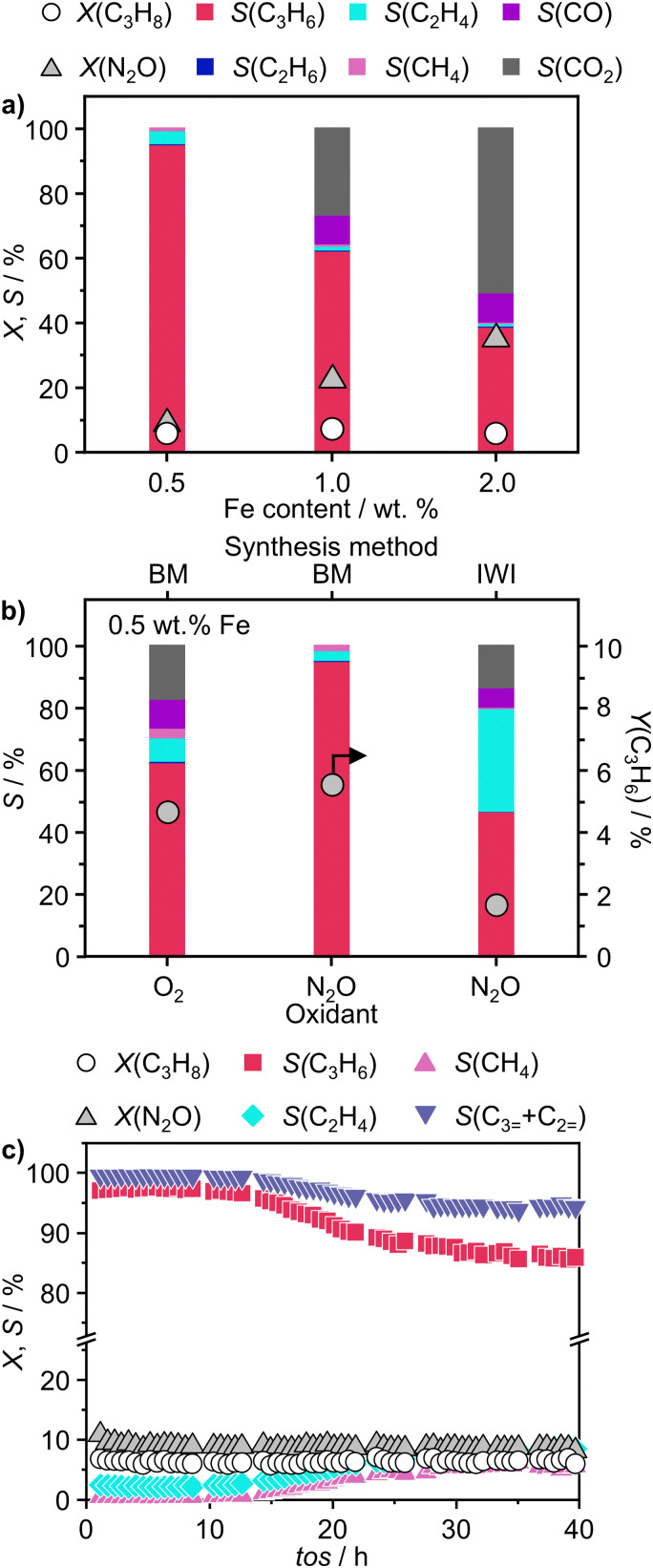
(a) N_2_O-ODHP performance of Fe/BN1 catalysts as a function of Fe content represented by product selectivity patterns and C_3_H_8_ and N_2_O conversion. (b) Product selectivity patterns and C_3_H_6_ yield of 0.5Fe/BN1 and 0.5Fe/BN-IWI in ODHP with O_2_ and N_2_O. (c) Stability test of 0.5Fe/BN1 in N_2_O-ODHP. Conditions: (a) *m*_cat_ = 0.05–1 g or (b) and (c) *m*_cat_ = 1 g; (all) *F*_T_ = 20 cm^3^ min^−1^; *T* = 723 K; feed = 8 vol% C_3_H_8_, 8 vol% N_2_O, 84 vol% He or 8 vol% C_3_H_8_, 4 vol% O_2_, 88 vol% He; *P* = 1 bar.

To validate the ability of the catalysts in selective oxidations without promoting coke formation, the catalysts were then tested in the nitrous oxide-mediated oxidative dehydrogenation of propane (N_2_O-ODHP). Recently, boron nitrides were identified as highly promising catalysts when using O_2_ as an oxidant (O_2_-ODHP), owing to the effective suppression of overoxidation phenomena at propane conversion below 5%.^1^ Notably, a control test revealed that pristine h-BN has negligible activity in the presence of N_2_O (Fig. S13, ESI†). This likely stems from the lower oxidising potential of N_2_O, which is not able to oxidise the surface of h-BN to generate active sites for further N_2_O activation and subsequent propane adsorption. Furthermore, it has been investigated whether catalytic activity may have been conferred to the material, *i.e.* BN1, during ball milling as a result of surface functionalization. However, propane conversion was observed only for temperatures above 773 K, as for pristine h-BN. The influence of these functional groups, as well as vacancies, appears to be relatively minor. This is evident when observing that the product distribution closely aligns with that obtained in the blank experiment, where the reactor was packed with quartz particles. In this respect, it must be noted that oxidative dehydrogenation reactions can be influenced by radical gas-phase activity.^74,75^ Thus, the observed activity for h-BN and BN1 at elevated temperatures may potentially originate from this phenomenon. From one perspective, the negligible activity of the supports can be regarded as a favourable outcome, in accordance with the objective of employing a support material that does not exhibit remarkable interactions with the reacting molecules. Initial activity tests of Fe/BN catalysts (Fig. S14 and S15, ESI†) showed that, especially for the mechanochemically-derived catalysts, *i.e.* Fe/BN1 with varying Fe content, operating at temperatures below 773 K effectively inhibits C–C bond-breaking products and slows overoxidation kinetics, resulting in low selectivity towards CO_*x*_. Most importantly, the near-total propylene selectivity achieved at near-zero propane conversion with 0.5Fe/BN1 strongly implies a notably slow kinetics for direct propane oxidation.^76^ After evaluating the temperature dependence of the product distribution, experiments at a fixed propane conversion level of 6% for Fe/BN1 catalysts were conducted (Fig. 4a). The performance of these catalysts showcased notable variations, which were attributed to differences in Fe species present. The Fe patches and NPs appeared to promote C–C bond cleavage and subsequent overoxidation resulting in the formation of CO_*x*_ byproducts. Conversely, Fe SAs demonstrated the ability to establish moderate interaction with the substrate, thereby selectively producing propylene, albeit with mild activity (Fig. S16, ESI†). The minor formation of byproducts, such as ethylene and methane, could be attributed to the presence of sporadic low nuclearity clusters in 0.5Fe/BN1. Most importantly, for the latter catalyst, the reduction in weight-hour-space-velocity (*WHSV*), achieved by increasing the catalyst mass in the reactor, did not noticeably affect the product distribution. Indeed, propylene selectivity remained consistent at higher propane conversions (*S*(C_3_H_6_) = 95% at *X*(C_3_H_8_) = 6%). This outcome strongly suggests that this material does not favour subsequent oxidation of the desired product.^76^ To stress the significance of the adopted synthesis methodology, the performance of 0.5Fe/BN1 and 0.5Fe/BN-IWI was assessed under the same operating conditions (Fig. 4b). Notably, the former allowed the attainment of a propylene yield exceeding that of the latter by a factor of two. This outcome primarily stems from the discrepancy in terms of selectivity to propylene. In the case of 0.5Fe/BN-IWI, the cleavage of C–C bonds was favoured. Here, the respective C_2_ fraction likely had sufficient energy to desorb, while the C_1_ counterpart remained adsorbed on the surface of the catalyst, undergoing overoxidation thus leading to the marked formation of CO_*x*_ products. The adoption of FeCl_3_ instead of the nitrate iron precursor resulted in worse performance (Fig. S17, ESI†), which can be attributed to a distinct nanostructure from the optimal sample (0.5Fe/BN1). Moreover, the role of the oxidant was considered by evaluating the performance of 0.5Fe/BN1 at identical conditions with the only variation being the oxidant employed, namely O_2_, keeping a constant number of oxygen atoms in the reactive atmosphere. It can be seen that despite the higher activity observed in the presence of O_2_, the propylene yield was lower compared to when N_2_O is used. This stems from the enhanced propylene selectivity attributable to the formation of selective oxygen species from N_2_O, consistent with prior findings.^77–79^ There, a mechanism for Fe-containing zeolites involving highly isolated extra framework Fe–O–Al sites as active centers has been proposed.^80–82^ The reaction initiates with N_2_O adsorption on Fe, releasing N_2_ and forming Fe–O species, with the oxidation state of iron influenced by the partial delocalization of the negative charge over the oxygen atom (Fe^2+^–O, Fe^3+^–O^−^). It is proposed that Fe^3+^–O^−^ interacts with either propane or propylene *via* an Eley Rideal (ER) mechanism, producing propylene and CO_*x*_, respectively. Our initial activity tests demonstrated negligible activity for pristine h-BN and BN1 compared to Fe-containing samples (Fig. S13 and S14, ESI†). Prior computational studies corroborate these findings, showing N_2_O adsorption energies of approximately −3.6 kcal mol^−1^,^83^*versus* −12.9 and −25 kcal mol^−1^ on Fe centers stabilized 
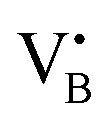
 and 
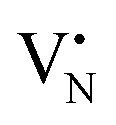
, respectively.^84^ Given the poor alkane adsorption properties of the BN surface, we infer that N_2_O adsorption on Fe centers leads to N_2_ evolution and Fe–O species formation, which primarily interact with propane to form propylene *via* an ER mechanism. However, when Fe_*x*_O_*y*_ species are present, a Mars–van Krevelen (MvK) mechanism becomes significant, leading to overoxidation and CO_*x*_ byproducts.^85,86^ Thus, a clear distinction on reaction pathways mediated by Fe SAs and NPs can be made. Future studies focusing on detailed kinetic and computational analyses, as well as *in situ* or *operando* characterization, will be required to further substantiate these considerations.

**Fig. 5:**
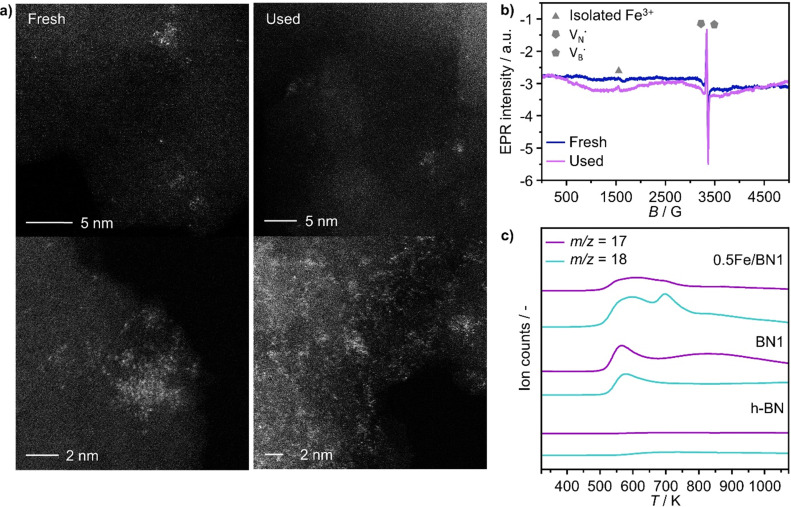
Characterization of 0.5Fe/BN1 before and after use in N_2_O-ODHP for 18 h. (a) High resolution HAADF-STEM micrographs and (b) EPR spectra acquired at 293 K of 0.5Fe/BN1 after the reaction. (c) NH_3_-TPD of as prepared 0.5Fe/BN1 and BN supports.

Finally, to assess the stability and potential deactivation phenomena, stability tests were conducted on selected catalysts at 723 K. Notably, for 0.5Fe/BN-IWI, a significant reduction in propane conversion, reaching 45% of the initial value, was observed after 15 h on stream (Fig. S18, ESI†). Conversely, the decline in N_2_O conversion was relatively milder, with only a 12% decrease compared to the initial value. This decrease in activity could be primarily attributed to the generation of larger NPs, resulting in a reduction of exposed surface area of the active phase. Moreover, CO_*x*_ production increased with a concomitant decrease in propylene selectivity, aligning with the previous findings on the structure sensitivity and thus the extent of the overoxidation phenomena. Conversely, 2Fe/BN1 exhibited consistent propane conversion, maintaining a level of approximately 15% for a duration of at least 12 h (Fig. S19, ESI†). However, the conversion of N_2_O displayed a decreasing trend, starting from *ca.* 50% and stabilising at approximately 37% after 8 h of stream. It is worth noting that propylene was the predominant olefin produced. The trend of propylene production also stabilised after approximately 8 h, coinciding with the equilibration of N_2_O conversion. This suggests the *in situ* formation of a composition that enhances the selective production of propylene, leading to an increase of approximately 30% compared to the initial value. Such increase in selectivity was accompanied by a reduction in the fraction of CO_*x*_ products, which justifies the decrease in N_2_O conversion. 0.5Fe/BN1 exhibited stable conversion for both N_2_O (*ca.* 9%) and C_3_H_8_ (*ca.* 6%) over 40 h on stream (Fig. 4c). The propylene and total olefin selectivity showed a gradual decrease followed by a stabilisation at approximately 86% and 94%, respectively. This outcome aligns with the consideration made on the reaction mechanism (*vide supra*). Indeed, the constant activity observed, likely due to the little amount of H_2_O produced, suggests that N_2_O activation occurs over Fe centers, where H_2_O forms and desorbs. This contrasts with the reaction and deactivation mechanism proposed for BN-based catalyst in O_2_-ODHP.^87,88^ Since this is the catalyst of main interest in this study, the sample was characterised after 18 h on stream, coinciding with the slight decrease in propylene selectivity to determine any changes in the nanostructure that may have contributed to this minor reduction in selectivity. Advanced microscopy showed no significant metal agglomeration with respect to the fresh sample (Fig. 5a and Fig. S20, ESI†). In addition, the used sample was analysed *via* EPR spectroscopy demonstrating the absence of any remarkable signals attributable to aggregates or NPs after 18 h on stream (Fig. 5b). However, based on the structure-sensitivity established in this study, the drop in selectivity may stem from the aggregation of unstable Fe SAs into small clusters. Moreover, since this reaction is known to be affected by coke formation and subsequent catalyst deactivation, a thermogravimetric analysis (TGA) was conducted, although the appearance of the catalyst did not change after the reaction. TGA revealed no loss in weight during the temperature ramp (Fig. S21, ESI†). Conversely, a slight weight increase was observed, which can be attributed to the complete oxidation of catalyst surface by O_2_ during the TGA measurement. Moreover, no signals assignable to carbenium radicals were observed by EPR. To elucidate the remarkable capacity of this catalyst to selectively trigger C–H bond activation, albeit with limited activity, while avoiding the formation of coke, the acidity of the catalyst surface was assessed using NH_3_ temperature-programmed desorption (NH_3_-TPD). In this regard, cationic species on the catalyst surface are known to induce extensive coking by participating in acid-catalysed carbenium ion reactions.^37^ This process involves substrate dehydrogenation followed by polycondensation.^89^ NH_3_-TPD for h-BN confirmed the complete absence of surface acidic sites (Fig. 5c). Note that both *m*/*z* 17 and 18 are depicted, as OH and NH_3_ may both contribute to the ion count at *m*/*z* 17. According to the mass spectrum of H_2_O, the contribution at *m*/*z* 17 exceeds 20% of that at *m*/*z* 18.^90^ Indeed, for BN1, the first peak for *m*/*z* 17 at 575 K may contain a component deriving from OH groups bound to the surface of the catalyst. Still, the major component of this peak derives from desorption of NH_3_. Thus, the mechanochemical treatment imparted a slight acidic character to BN1, although this effect was not pronounced. Conversely, the weak *m*/*z* 17 signal of 0.5Fe/BN1 is predominantly due to OH groups. This is evident as the poorly defined peaks in the *m*/*z* 17 signal at approximately 600 and 700 K coincide with the well-defined peaks for *m*/*z* 18. Therefore, this observation confirms the non-acidic nature of 0.5Fe/BN1. Intriguingly, comparison of spectra between this catalyst and BN1 revealed the presence of two distinct OH sites on the catalyst surface. The desorption at lower temperature likely corresponds to OH groups bound to the support, while the peak at higher temperatures may suggest the presence of OH ligands on Fe. Hence, NH_3_-TPD demonstrated the lack of significant acidic sites on 0.5Fe/BN1 allowing selective C–H bond activation without promoting coke formation. These findings highlight the crucial capability of the mechanochemical activation in stabilising Fe SAs on a host devoid of remarkable protonic sites without altering the acidic nature of the support.

### Catalyst benchmarking

**Fig. 6:**
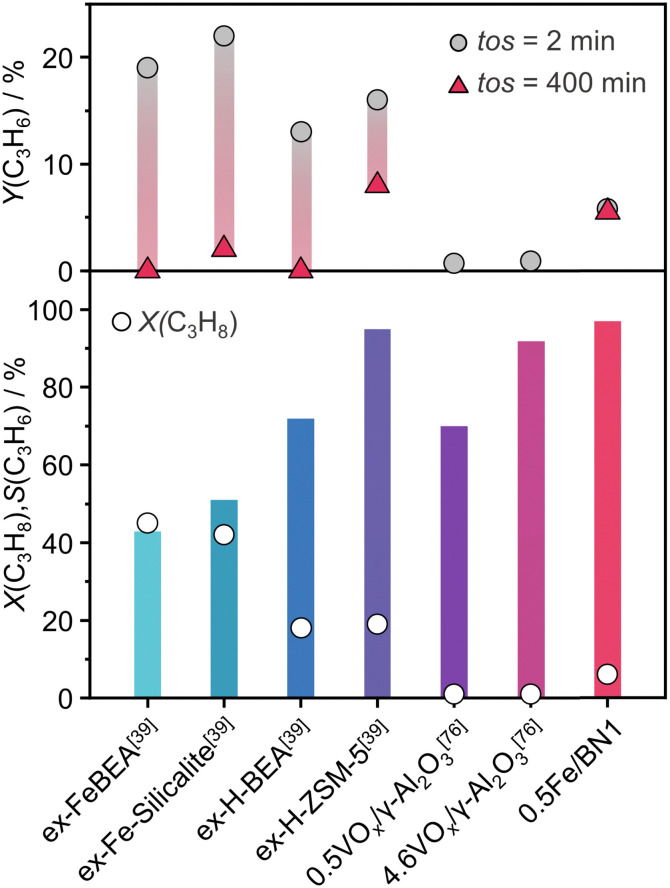
N_2_O-ODHP performance, expressed in terms of C_3_H_8_ conversion, C_3_H_6_ selectivity and yield, of benchmark catalysts reported in the literature. The reaction conditions are provided in Table S3 (ESI†).

The mechanochemical activation in the ball mill resulted in the development of a novel and promising catalytic system (*i.e.*, 0.5Fe/BN1) for C–H bond activation in presence of N_2_O. To gain a broader perspective on the performance of this catalyst at relevant N_2_O-ODHP conditions (*T* = 723 K and *P* = 1 bar), we compared it with the established benchmark systems tested in N_2_O-ODHP, comprising Fe-zeolites,^39^ as well as both crystalline and amorphous VO_*x*_ species supported on γ-Al_2_O_3_.^76^ Several performance metrics were considered for the comparison, including the highest achieved C_3_H_6_ selectivity with the respective C_3_H_8_ conversion, and the initial and final C_3_H_6_ yields corresponding to a time-on-stream (*tos*) of 2 and 400 min, respectively (Fig. 6).

Interestingly, in each of the aforementioned benchmark systems, the support had a strong influence on performance. In the case of VO_*x*_, these species were unable to entirely cover the surface of the support, leading to inability to attain high propylene selectivity at appreciable propane conversion, since γ-Al_2_O_3_ can overoxidise propylene. This detrimental characteristic was notably absent in 0.5Fe/BN1, enabling superior propylene selectivity at higher degrees of propane conversion. Nonetheless, the catalytic activity of the system under investigation was lower than that observed in Fe-containing zeolites. Although the latter system was very active, cationic species exposed on the surface of the zeolite support promote coking leading to rapid deactivation and limiting the applicability of these catalysts. The clear discrepancy in the acidic nature (Fig. S22, ESI†) between Fe-containing zeolites and 0.5Fe/BN1 allowed the latter to remain the sole active and selective catalyst after 400 min on stream. This evaluation underscores the superior stability and selectivity of 0.5Fe/BN1 for propylene production *via* N_2_O-ODHP. Finally, these findings shed light on the potential of stabilising metal species on hosts devoid of acidic properties, which are of relevance to selective oxidations at large and encourage future investigations for novel h-BN-supported SACs.

## Conclusions

In summary, we showed the viability of mechanochemical activation for enabling atomic-scale control over the nanostructure of h-BN-based catalyst, offering practical guidelines for SACs design. In particular, the milling time and the metal content were found to be key descriptors to finely tune the host's structural properties such as surface area, oxygen content and defect density. These features played a crucial role in stabilising Fe centres over the carrier, emphasising the critical interplay between mechanical forces and elevated local temperatures during ball milling, facilitating B–N bond cleavage, and the tendency of Fe to hinder such process, preserving the original crystal order of the host. Employing a low metal content (0.5 wt%), a mixture of Fe^2+^ and Fe^3+^ SAs was stabilised over the surface defects generated during the milling treatment. 0.5Fe/BN1 showcased efficient C–H activation in N_2_O-ODHP for selective and stable propylene production, while inhibiting coke formation and mitigating substrate overoxidation. For the latter phenomenon, the degree of Fe dispersion was found to be a relevant predictor of catalyst selectivity, wherein Fe agglomerates and NPs promoted the formation of CO_*x*_ and C–C bond breaking byproducts. Finally, the deposition of Fe sites on the host devoid of remarkable acidic sites revealed to be essential for hindering coking, ensuring stable operation over 40 h. This work underscores the potential of targeted catalyst engineering for unlocking selective catalytic pathways and mechanochemical activation as an effective synthetic methodology for the synthesis of h-BN-supported transition metal SACs, encouraging studies to explore their potential in selective oxidations at large.

## Author contributions

The manuscript was written through contributions of all authors. All authors have given approval to the final version of the manuscript. G. B. and I. S. have contributed equally.

## Data availability

The data that support the findings of this study are openly available in Zenodo at (https://doi.org/10.5281/zenodo.11520887), reference number 11520887. Further data supporting the findings of this study are available in the ESI.† All other relevant source data are available from the corresponding author upon request.

## Conflicts of interest

The authors declare no conflict of interest.

## Supplementary Material

EY-002-D4EY00123K-s001
