# Supplementary material for: Mechanochemically-derived iron atoms on defective boron nitride for stable propylene production
Source: EES Catal. 2024 Aug 7;2(6):1263–76. doi: 10.1039/d4ey00123k (PMC11320177; doi:10.1039/d4ey00123k)
Supplement: EY-002-D4EY00123K-s001 [file EY-002-D4EY00123K-s001.pdf]

Supplementary Information (SI) for EES Catalysis.
This journal is © The Royal Society of Chemistry 2024

Electronic Supplementary Information

Mechanochemically-derived iron atoms on defective boron nitride for stable propylene production

*Gian M. Beshara[+], Ivan Surin[+], Mikhail Agrachev, Henrik Eliasson, Tatiana Otroshchenko, Frank Krumeich, Rolf Erni, Evgenii V. Kondratenko, and Javier Pérez-Ramírez**

[+] Equal contribution.

* Corresponding author. E-mail address: jpr@chem.ethz.ch

Catalyst Synthesis. A series of *m*Fe/BN*n* catalysts and metal-free BN*n* supports were synthesized *via* ball milling (*m* = metal content in wt % and *n* = milling time in h) using a Retsch Mixer mill MM 500 Nano, with a nominal Fe content varying in the range of 0.5-2 wt %. The support, hexagonal-boron nitride (h-BN, Thermo Scientific, 99.5% metal basis) and the metal precursor, $Fe(NO_3)_3\cdot 9H_2O$ (Sigma-Aldrich, >98%), were loaded in a 50 cm^3 zirconium oxide milling jar with 16 zirconium oxide balls (diameter = 10 mm). A programmed cycle was developed, consisting of an initial phase lasting 30 s with a vibration frequency of 5 Hz followed by the main phase with a duration of 10 min and a frequency of 30 Hz. The total number of cycles was determined based on the desired duration of the ball milling process. Following the same procedure, two samples using anhydrous $FeCl_3$ (abcr, >98%) as metal precursor were synthesized, denoted with Cl as subscript of Fe ($m\mathrm{Fe_{Cl}}$/BN*n*).

A series of *m*Fe/BN-IWI catalysts were synthesized *via* incipient wetness impregnation (IWI) method with a nominal Fe content varying in the range of 0.5-2 wt %. Accordingly, appropriate amounts of $Fe(NO_3)_3\cdot 9H_2O$ (Sigma-Aldrich, >98%) were dissolved in deionized water and the resulting solutions were added dropwise to the support. Following the impregnation step, the samples were dried overnight under vacuum at 353 K and then calcined in static air at 873 K (heating rate = 5 K min^{-1}, dwell time = 5 h). The synthesis of 0.5Fe/BN1-IWI and 0.5Fe/BN1-IWI-N_2 followed the same procedure, using BN1, *i.e.*, h-BN ball-milled for 1 h as the support. These samples were subsequently annealed in static air and N_2 atmosphere, respectively.

Commercial ZSM-5 zeolite with nominal Si/Al molar ratio of 40 (CBV 8014) was supplied by Zeolyst International in the ammonium form. The respective protonated form, H-ZSM-5, was obtained by calcining the ammonium form in static air at 823 K (heating rate = 5 K min^{-1}, dwell time = 5 h).

Catalyst Characterization. The metal content in the catalysts was measured by inductively coupled plasma optical emission spectroscopy (ICP-OES) using a Horiba Ultra 2 instrument equipped with a photomultiplier tube detector. Approximately 15 mg of the solid sample was loaded into a polytetrafluoroethylene tube along with 3 cm^3 of HNO_3. The tubes were then subjected to microwave irradiation, raising the temperature to 533 K, and maintained at autogenous pressure of 40 bar for 20 min. After digestion, the resulting clear solutions were diluted to 25 cm^3 with millipore water and filtered prior to analysis.

Powder X-ray diffraction (XRD) was performed using a Rigaku SmartLab diffractometer with a D/teX Ultra 250 detector using Cu-Kα radiation (λ = 0.1541 nm) and operating in the Bragg–Brentano geometry. The data were acquired in the 10-70° 2θ range with an angular step size of 0.025° and a counting time of 1.5 s per step. The following references were utilized to compare the XRD pattern of the analyzed samples with potential phases contained within them: Fe 96-900-0666 and h-BN 96-900-8998. The relative crystallinity of the samples was determined from the ratio of the intensity of the (002) reflection of the h-BN lattice and that of the (116) reflection of α-Al_2O_3 (Alfa Aesar, 99.98%), which was well mixed with h-BN or BNn powder (h-BN:α-Al_2O_3 mass ratio = 7:4) as an internal reference. The values are reported with respect to the pristine h-BN, which was assigned 100% crystallinity. The peak shift of the (002) reflection of the h-BN lattice for mFe/BNn catalysts was evaluated after correcting for sample displacement with α-Al_2O_3 as internal standard.

N_2 isotherms were measured at 77 K using a Micromeritics TriStar II analyzer. Prior to the measurements, the samples (*ca.* 0.15 g) were degassed at 473 K under vacuum for 12 h. The Brunauer-Emmet-Teller (BET) method was used to determine the total surface area (S_{BET}). The pore volume (V_{pore}) was determined using Barret-Joyner-Halenda method on the adsorption isotherm.

Scanning transmission electron micrographs (STEM) using a high-angle annular dark-field (HAADF) detector were captured using an aberration corrected HD2700CS Hitachi microscope operating at 200 kV. To prepare the samples for imaging, a copper grid with a perforated carbon foil was dipped into an ethanol suspension containing the solid powder. Subsequently, the sample was dried in ambient air and gently cleaned with an argon-oxygen plasma to reduce sample contamination. HAADF-STEM images and energy dispersive X-ray spectroscopy (EDXS) mappings of as-prepared and used 0.5Fe/BN1, presented in **Figure 5** of the manuscript, were acquired with a probe aberration-corrected Titan Themis operated at 300 kV, equipped with a Super-X EDX detector.

X-ray photoelectron spectroscopy (XPS) was performed on a Physical Electronics Quantum 2000 spectrometer using monochromatic Al-Kα radiation, generated by an electron beam operated at 15 kV, and equipped with a hemispherical capacitor electron-energy analyzer. The samples were analyzed at an electron take-off angle of 45° and a constant analyzer pass energy of 46.95 eV with a spectra resolution step width of 0.2 eV.

Continuous wave-electron paramagnetic resonance (CW-EPR) spectroscopy experiments were conducted on a Bruker Elexsys E500 spectrometer operating at X-band frequencies, using an ER4102ST microwave resonator and equipped with an Oxford helium (ESR900) cryostat. All CW-EPR spectra were acquired at room temperature and 10 K with the following spectrometer parameters: microwave frequency = 9.4 GHz, sweep width = 499 mT, center field = 250 mT, modulation frequency = 100 kHz, modulation amplitude = 3 G, microwave power = 2.012 mW, power attenuation = 20 dB, conversion time = 327.68 ms, time constant = 81.92 ms. All measured g-factors were offset-corrected against a known standard (*i.e.*, free radical 1,1-diphenyl-2-picrylhydrazyl). The spectral simulations were performed by using the Easyspin toolbox, implemented on MATLAB.

Ultraviolet-visible (UV-vis) spectroscopy was conducted using an Avantes AVASPEC fiber optic spectrometer fitted with an AvaLight-DH-S-BAL deuterium-halogen light source and a CCD array detector. $BaSO_4$ served as the white reference material. The catalyst (m_{cat} = 0.1 g, powder) was loaded into a quartz reactor (inner diameter = 6 mm). The catalyst bed was fixed using two layers of quartz wool. A high-temperature reflection probe, comprising six light fibers and one reading fiber, was positioned perpendicular to the reactor. The catalyst was heated to 723 K in flowing N_2 (total volumetric flow, F_T = 10 cm^3 min^{-1}, heating rate = 10 K min^{-1}, hold time = 15 min) prior to spectra acquisition.

Raman spectroscopy was performed on a Horiba LabRAM HR Evolution UV-vis-NIR confocal Raman system using a Cobolt Samba Nd/YAG laser with a wavelength of 532 nm, a power of 5.7 mW and a 50× Olympus LMPlanFLN objective. Spectra were collected with an acquisition time of 10 s and an accumulation number of 5. The spectra depicted in **Figure 2c** and **Figure S4** were obtained by subtracting a flat baseline.

Fourier transform infrared spectroscopy (FTIR) was conducted using a Bruker Optics Vertex 70 spectrometer equipped with a custom-built transmission cell featuring KBr windows and a liquid-nitrogen-cooled mercury cadmium telluride (MCT) detector. Catalyst wafers were prepared by subjecting them to a pressure of 3.85×10^7 kg m^{-2} for 20 s and subsequently degassed at 10^{-6} bar and 723 K for 4 h prior to analysis. FTIR spectra were obtained by averaging 64 scans over the range of 650-4000 cm^{-1}, with a nominal spectral resolution of 4 cm^{-1}. Each spectrum reported in **Figure 2d** was normalized.

Thermogravimetric analysis (TGA) profiles were acquired using a Linseis STA PT1600 system. The sample (20 mg) was dried in 20 vol% O_2 in N_2 at 353 K (F_T = 100 cm^3 min^{-1}, heating rate = 20 K min^{-1}, hold time = 90 min). Subsequently the temperature was increased from 353 to 1073 K (heating rate = 5 K min^{-1}, hold time = 60 min) to quantify the amount of coke in the used catalysts.

Temperature-programmed desorption of ammonia (NH_3-TPD) was carried out using a Micromeritics AutoChem HP II analyzer equipped with a TCD and coupled to a Pfeiffer Vacuum OmniStar MS. Prior to NH_3-TPD measurements, samples (0.2 g) were loaded into a quartz tube, dried under flowing He at 423 K for 1 hour (F_T = 20 cm^3 min^{-1}, heating rate = 10 K min^{-1}), and then cooled down to 313 K (cooling rate = 20 K min^{-1}). After 10 min, the sample was saturated under flowing 10 vol% NH_3 in He (F_T = 20 cm^3 min^{-1}) for 1 h. The initial desorption of weakly bound NH_3 was conducted at 473 K under flowing helium (F_T = 20 cm^3 min^{-1}) for 20 min. Temperature-programmed desorption was then performed under flowing He (F_T = 20 cm^3 min^{-1}) in the range of 313–1073 K (heating rate = 10 K min^{-1}), with NH_3 evolution monitored by the TCD and MS.

Catalyst Evaluation. The oxidative dehydrogenation of propane was performed at atmospheric pressure in a PID Eng&Tech Microactivity Effi continuous-flow setup (**Figure S1**). The gases, C_3H_8 (Linde, purity 3.5), N_2O (40 vol% in He, Messer, purity 5.0) and He (PanGas, purity 5.0, carrier gas), were fed using digital mass-flow controllers (Bronkhorst) to the mixing unit. A quartz micro-reactor of 9.3 mm inner diameter was loaded with the catalyst (m_{cat} = 0.05-1 g for initial catalytic activity tests and 1 g for stability tests) held in place by a quartz wool bed set on a quartz frit and placed in the furnace of the setup. During the reaction, the temperature was controlled using a K-type thermocouple fixed in a coaxial quartz thermowell with the tip positioned in the center of the catalyst bed. Prior to testing, the undiluted catalyst was heated by reaction feed mixture (8 vol% C_3H_8, 8 vol% N_2O, and 84 vol% He) with a total volumetric flow of F_T = 20 cm^3 min^{-1} to the desired temperature and allowed to stabilize for at least 30 min before starting the measurements. The outlet stream was quantified online *via* a gas chromatograph equipped with CP7554PT PoraPLOTQ with two particle traps and Shin Carbon ST columns, and coupled to a thermal conductivity (TCD) and a flame ionization detector (FID). He, CO, CO_2 and N_2O signals were detected on TCD chromatograms, while hydrocarbons were detected on FID

chromatograms. The main reaction products were propylene (C_3H_6) and ethylene (C_2H_4), while carbon oxides (CO_x), methane (CH_4) and ethane (C_2H_6) were the side products.

The conversion of propane, X (C_3H_8), was calculated according to **Equation 1**:

$$X(\mathrm{C_3H_8}),\% = \frac{n_{\mathrm{C_3H_8}}^{\mathrm{inlet}} - n_{\mathrm{C_3H_8}}^{\mathrm{outlet}}}{n_{\mathrm{C_3H_8}}^{\mathrm{inlet}}} \times 100 \qquad (1)$$

where $n_{\mathrm{C_3H_8}}^{\mathrm{inlet}}$ and $n_{\mathrm{C_3H_8}}^{\mathrm{outlet}}$ are the molar flows of C_3H_8 at the reactor inlet and outlet, respectively. The selectivity, $S(j)$, to product j (j: C_3H_6, C_2H_4, C_2H_6, CO and CO_2) was obtained according to **Equation 2**:

$$S(j),\% = \frac{n_j^{\mathrm{outlet}}}{\sum n_j^{\mathrm{outlet}}} \times 100 \qquad (2)$$

where n_j^{outlet} is the molar flow of carbon product j at the reactor outlet and the sum at the denominator is extended to the molar flows of all carbon-containing products out of reactor. The propylene yield was calculated according to **Equation 3**:

$$Y(\mathrm{C_3H_6}),\% = \frac{X(\mathrm{C_3H_8}) \times S(\mathrm{C_3H_6})}{100} \qquad (3)$$

The carbon balance was determined according to **Equation 4**, and was usually closed above 95%:

$$\varepsilon_{\mathrm{C}},\% = \frac{n_{\mathrm{C_3H_8}}^{\mathrm{outlet}} + \sum_{\mathrm{j}} n_{\mathrm{j}}^{\mathrm{outlet}} \times \frac{N_{\mathrm{C},j}}{3}}{n_{\mathrm{C_3H_8}}^{\mathrm{inlet}}} \times 100 \qquad (4)$$

where $N_{\mathrm{C},j}$ is the number of carbon atoms in product j.

Table S1. Synthesis conditions and characterization data of the catalysts.

Catalyst	Preparation method[a]	Milling time / h	V_{pore}[b] / $cm^3\ g^{-1}$	S_{BET}[b] / $m^2\ g^{-1}$	Fe content[c] / wt.%
h-BN	-	-	0.05	14	-
BN0.5	BM	0.5	0.21	71	-
BN1	BM	1	0.33	136	-
BN2	BM	2	0.38	268	-
BN3	BM	3	0.43	392	-
0.25Fe/BN1	BM	1	0.20	62	0.19
0.5Fe/BN1	BM	1	0.11	44	0.45
0.5Fe/BN1-18h	BM	1		43	-
1Fe/BN1	BM	1	0.09	39	0.83
2Fe/BN1	BM	1	0.05	26	2.00
0.5Fe_{Cl}/BN1	BM	1	0.21	81	-
0.5Fe_{Cl}/BN2	BM	2	0.36	241	-
0.5Fe/BN-IWI	IWI	-	0.05	13	0.41
1Fe/BN-IWI	IWI	-	-	15	0.90
2Fe/BN-IWI	IWI	-	0.08	17	1.71
0.5Fe/BN1-IWI	IWI	1	0.23	55	-
0.5Fe/BN1-IWI-N_2 [d]	IWI	1	0.23	71	-

[a] BM: ball milling, IWI: incipient wetness impregnation; [b] N_2 sorption; [c] ICP-OES; [d] Annealing in N_2 atmosphere.

Table S2. Summary of the position and the full width at half maximum (*FWHM*) of the E_{2g} band detected by Raman spectroscopy, showing notable changes with milling time and Fe content. In samples without Fe, *FWHM* increased gradually with longer milling time, evidencing the introduction of defects within the horizontal plane. Conversely, for a fixed milling time of 1 h, the addition of Fe led to a decrease in *FWHM*, indicating the tendency of Fe atoms to preserve the original crystal structure of pristine h-BN. Additionally, a discernible blue shift from the pristine h-BN bulk peak was observed in milled samples, providing further evidence for the gradual exfoliation of h-BN - this process was hindered in Fe-containing samples as Fe content increased.

Catalyst	Position / cm^{-1}	*FWHM* / cm^{-1}
h-BN	1365.6	9.3
BN0.5	1365.8	10.8
BN1	1365.9	13.8
BN2	1366.4	14.8
BN3	1367.3	19.7
0.25Fe/BN1	1365.7	12.5
0.5Fe/BN1	1365.8	11.8
1Fe/BN1	1365.3	11.7
2Fe/BN1	1365.5	9.7

Table S3. Summary of the reaction conditions and catalytic performance metrics of N_2O-ODHP catalysts reported in the literature and in this study.

Catalyst	T / K	Molar C_3H_8:N_2O / -	$X(C_3H_8)$ / %	$S(C_3H_6)$ / %	$Y_0(C_3H_6)^a$ / %	$Y_f(C_3H_6)$ / %	Ref.
ex-Fe-BEA	723	1	45	43	19	0	39
c-Fe-Silicalite	723	1	14	48	5	0	39
ex-H-BEA	723	1	18	72	13	0	39
ex-H-ZSM-5	723	1	19	95	16	8	39
0.5VO_x/γ-Al_2O_3	723	1	1	70	1	-	76
4.6VO_x/γ-Al_2O_3	723	1	1	92	1	-	76
0.5Fe/BN1	723	1	6	95	6	6	This work

[a] Y_0 and Y_f refer to the activity after 2 and 400 min on stream, respectively.

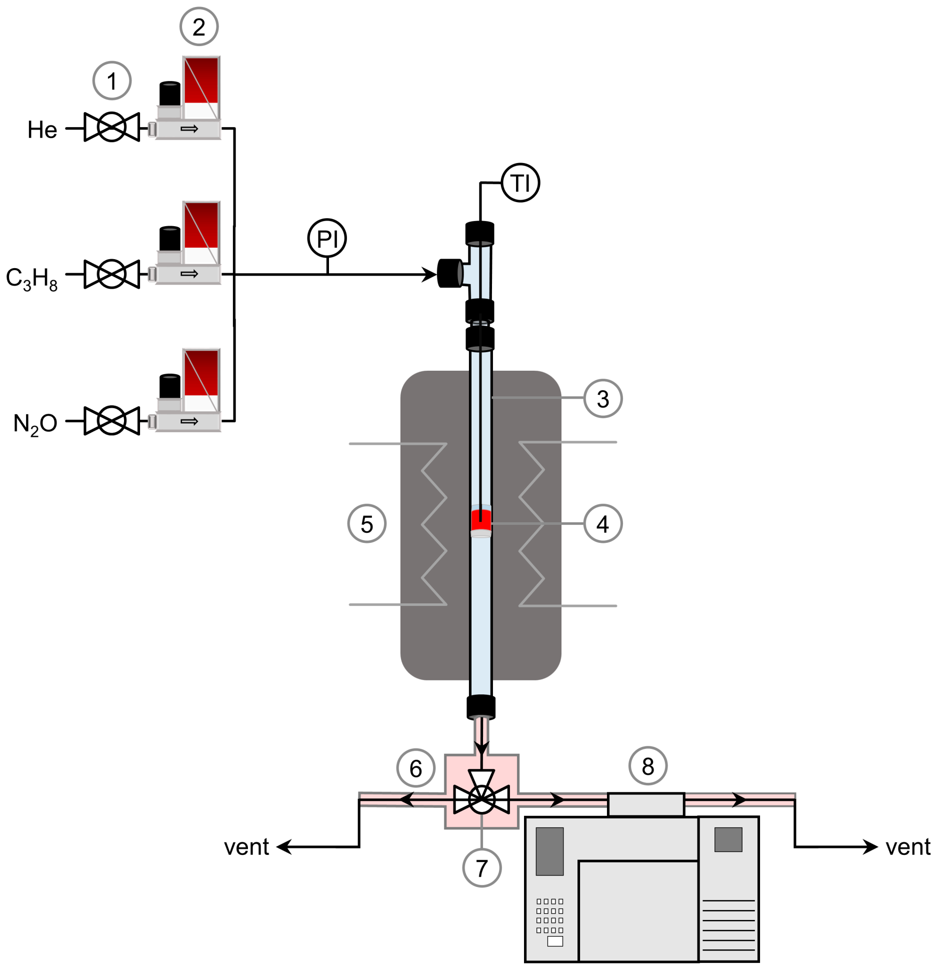

Figure S1. Scheme of the laboratory set-up used for oxidative propane dehydrogenation. 1: two-way on/off valves, 2: mass flow controllers, 3: quartz reactor, 4: catalyst bed, 5: oven, 6: heat tracing (red background), 7: three-way sampling valve, 8: gas chromatograph, PI: pressure indicator, and TI: temperature indicator.

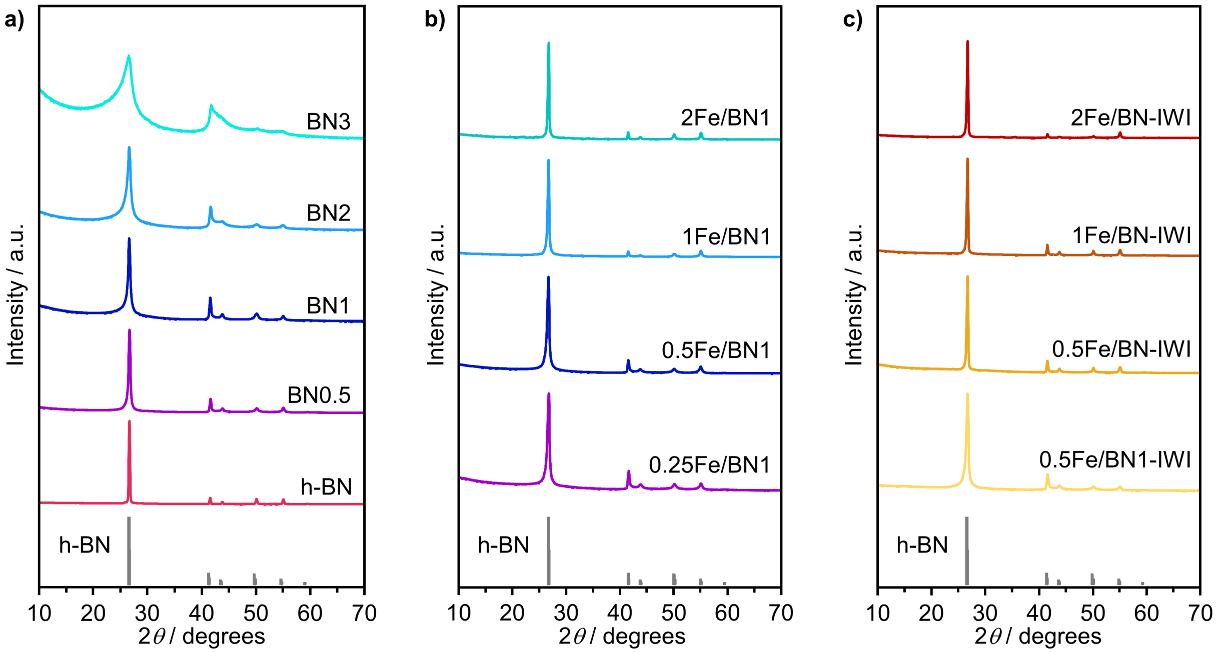

Figure S2. XRD patterns of as-prepared **a)** ball-milled h-BN samples after different milling times; **b)** ball-milled samples after a fixed milling time of 1 h and varying Fe content; **c)** IWI samples with varying Fe content.

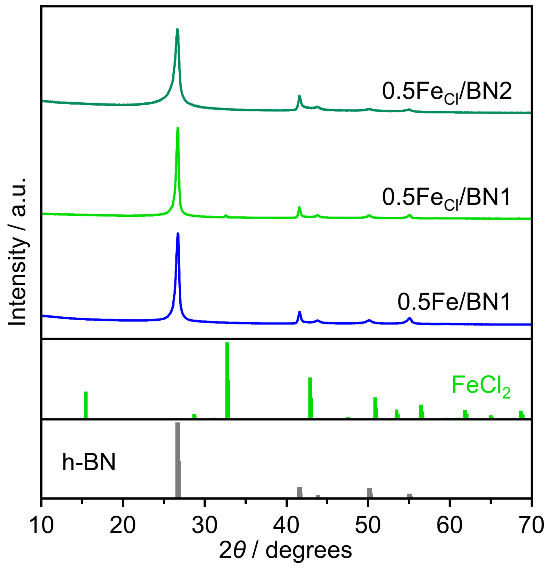

Figure S3. XRD patterns of as-prepared milled sample using different metal salt precursors and milling.

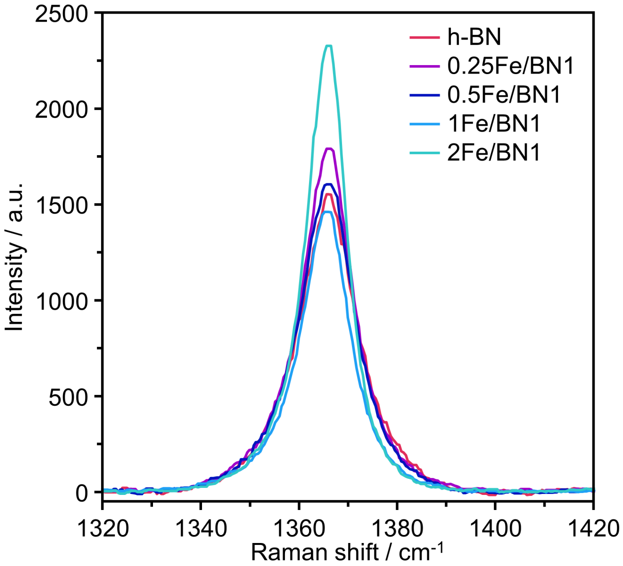

Figure S4. Raman spectra of as-prepared Fe/BN catalysts. Raman band at 1365 cm^{-1} represents the E_{2g} phonon vibrational mode originating from the in-plane vibration of B and N atoms in opposite directions. Generally, the *FWHM* decreases with increasing Fe content in the catalyst, suggesting that the metal hinders the introduction of disorder in the basal plane of h-BN (**Table S2**).

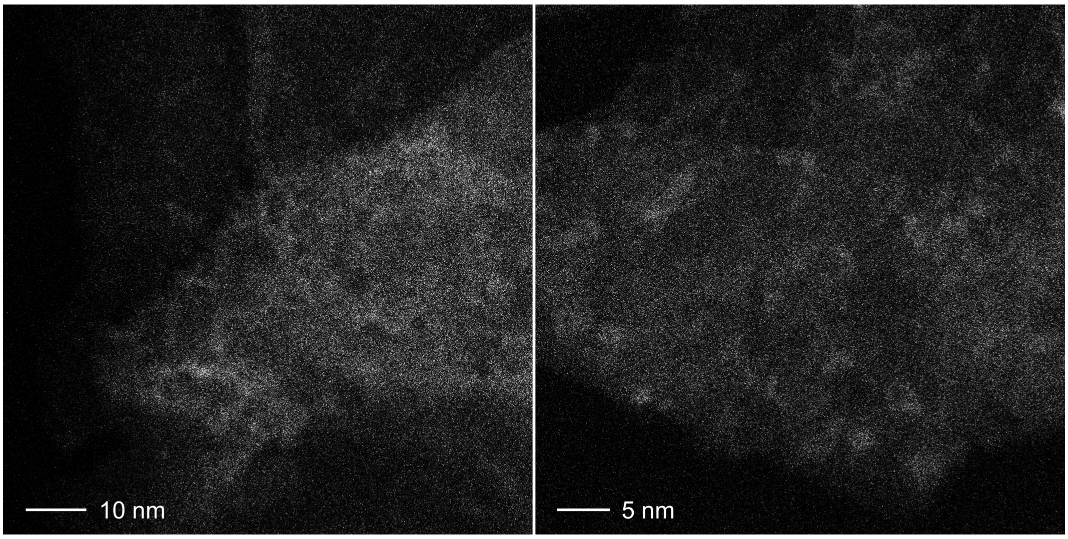

Figure S5. HAADF-STEM micrographs of as-prepared 2Fe/BN1 that illustrate the presence of small clusters and atomically-dispersed iron species.

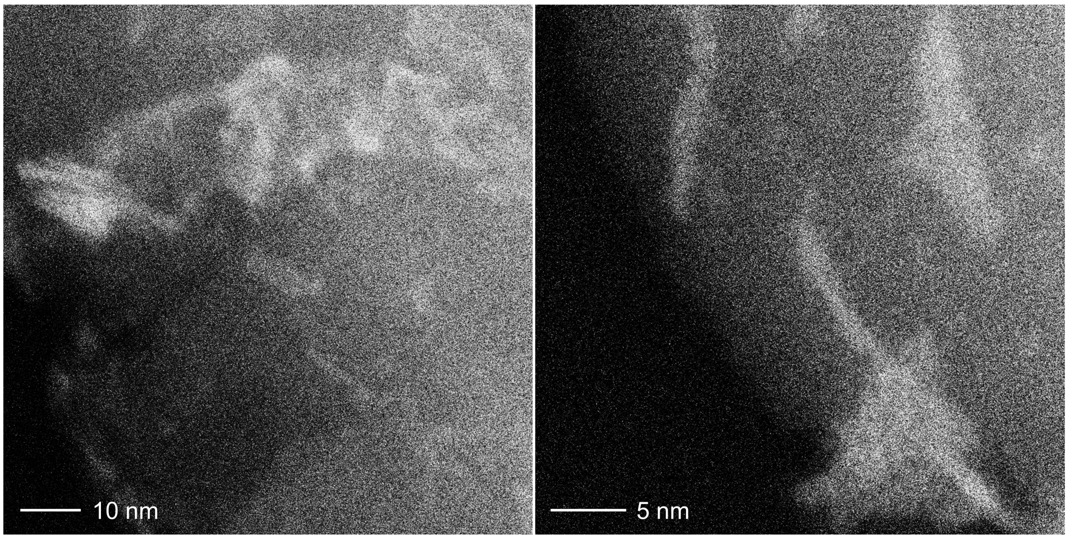

Figure S6. HAADF-STEM micrographs of as-prepared 1Fe/BN1 that illustrate the presence of Fe-enriched areas as patches.

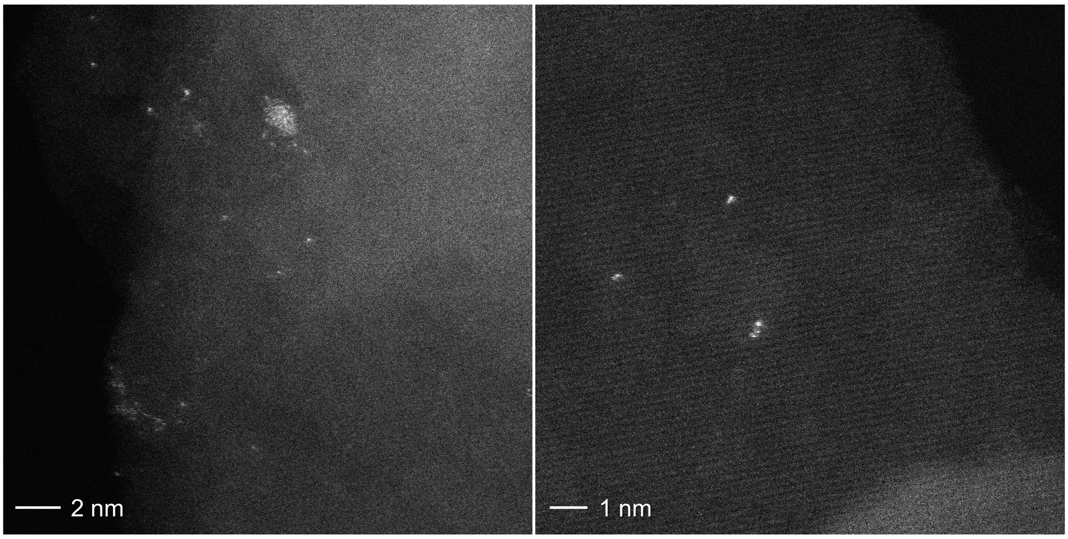

Figure S7. HAADF-STEM micrographs of as-prepared 0.5Fe/BN1 that illustrate the presence of atomically-dispersed iron species and eventually clusters.

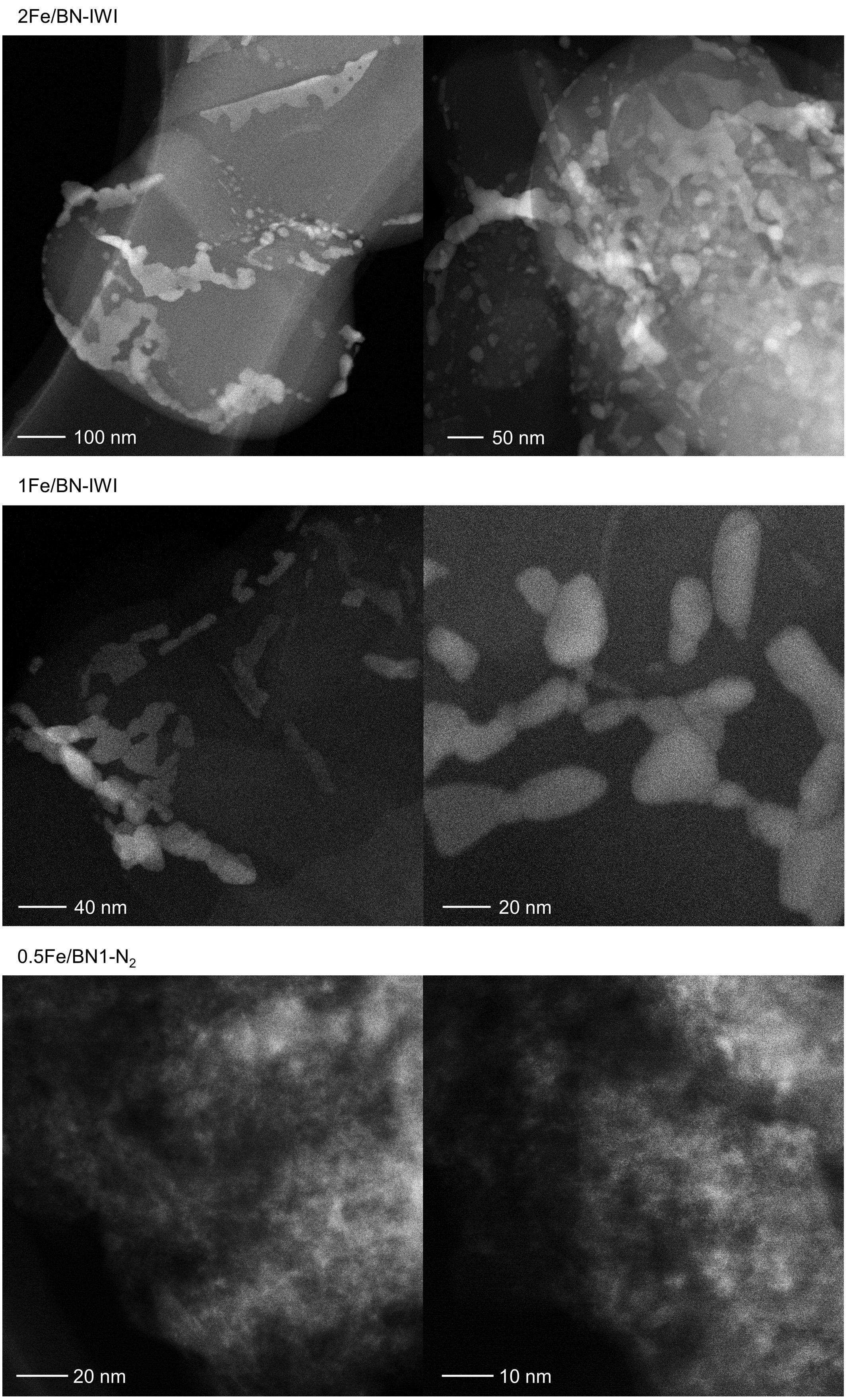

Figure S8. HAADF-STEM micrographs of as-prepared Fe/BN-IWI samples with different Fe content and supports.

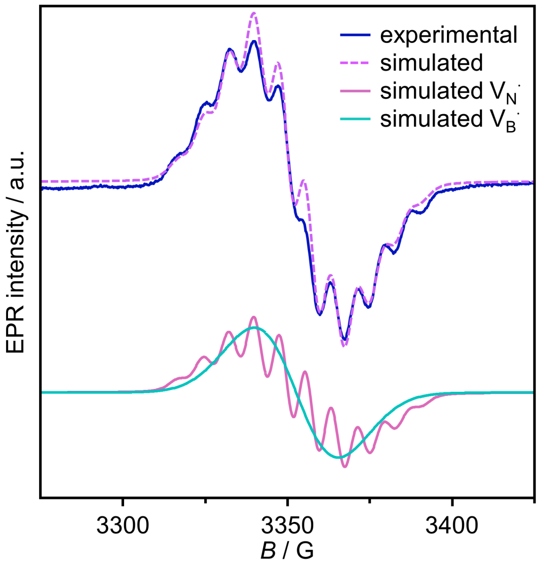

Figure S9. Experimental EPR spectrum of 0.5Fe/BN1 and simulated signal evidencing the presence of N and B vacancies ($V_N^{\cdot}$ and $V_B^{\cdot}$). The simulated signal represented by the dashed line was obtained by overlapping the two simulated signals corresponding to $V_N^{\cdot}$ and $V_B^{\cdot}$.

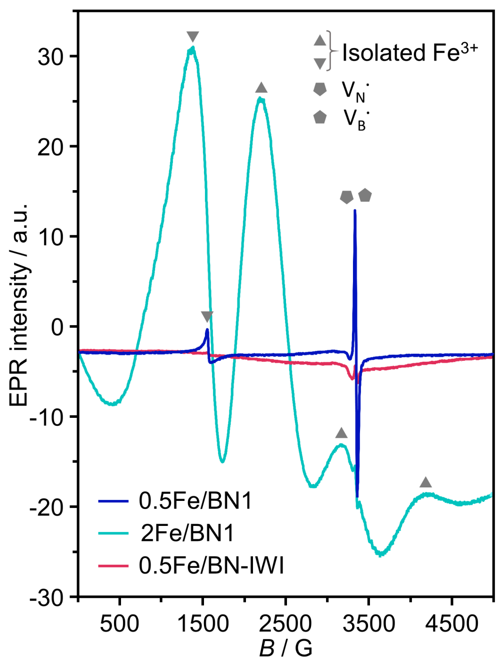

Figure S10. EPR spectra of selected as-prepared Fe/BN catalysts acquired at 10 K. Different symbols used for isolated paramagnetic Fe^{3+} sites correspond to species in different coordination environments, while $V_N^{\cdot}$ and $V_B^{\cdot}$ represent the nitrogen and boron vacancies, respectively. The low-temperature measurement amplifies the signal of isolated Fe^{3+} corroborating the presence of a minor fraction of Fe^{3+} in a distorted orthorhombic geometry in 0.5Fe/BN1 as shown by UV-vis spectroscopy in **Figure 3**.

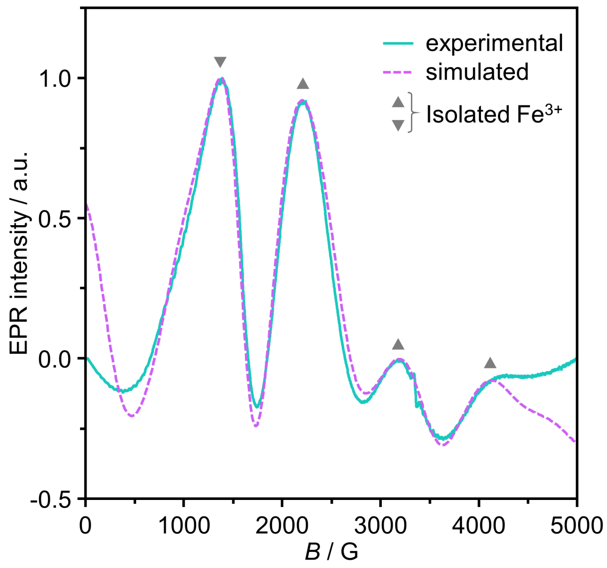

Figure S11. Experimental EPR spectrum of 2Fe/BN1 and simulated signal evidencing the presence of two different isolated iron sites. The inverted triangle indicates magnetically isolated Fe^{3+} in a highly distorted orthorhombic site while the upright triangle indicates a less distorted Fe^{3+} site.

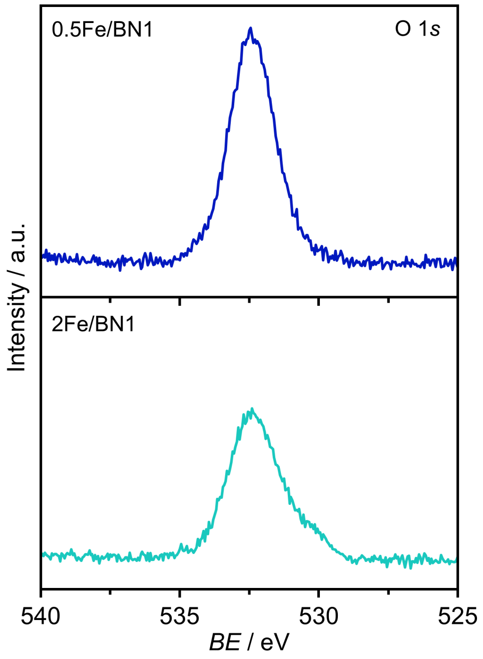

Figure S12. O 1*s* XPS spectra of selected as-prepared Fe/BN catalysts, evidencing the presence of oxygen due to the functionalization of the catalyst surface during the mechanochemical activation.

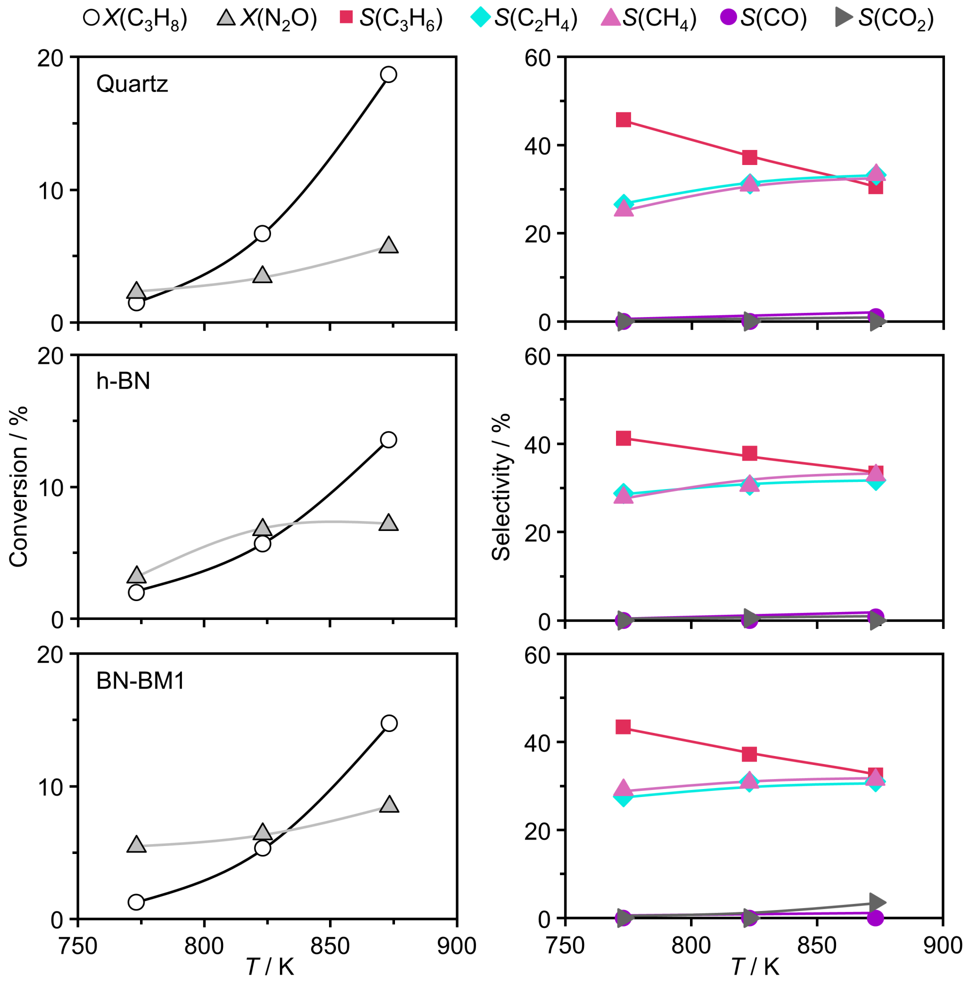

Figure S13. N_2O-ODHP performance of reference materials, expressed in terms of reactants conversion and product selectivity, as a function of reaction temperature. Conditions: T = 673-873 K; m_{cat} = 0.2 g; F_T = 20 cm^3 min^{-1}; Feed = 8 vol% C_3H_8, 8 vol% N_2O, 84 vol% He; P = 1 bar.

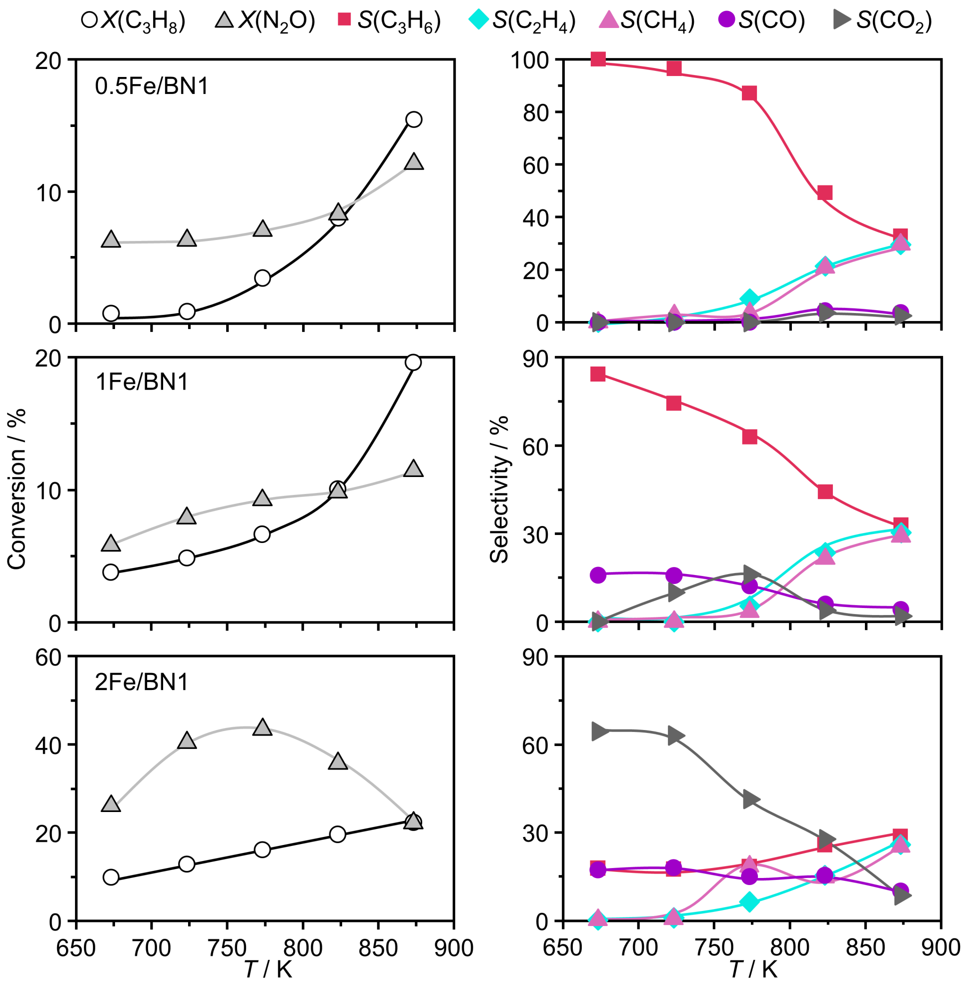

Figure S14. N_2O-ODHP performance of Fe/BN1 catalysts with varying Fe content, expressed in terms of reactants conversion and product selectivity, as a function of reaction temperature. Conditions: T = 673-873 K; m_{cat} = 0.2 g; F_T = 20 cm^3 min^{-1}; Feed = 8 vol% C_3H_8, 8 vol% N_2O, 84 vol% He; P = 1 bar.

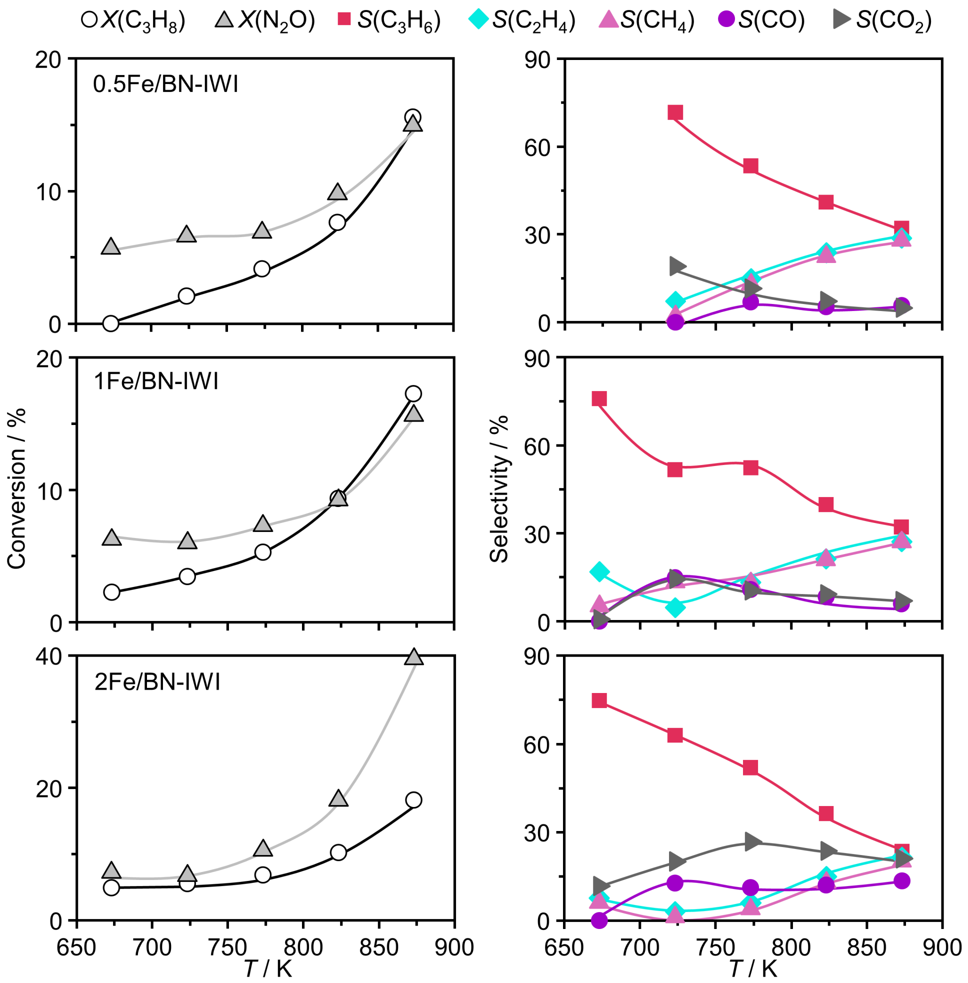

Figure S15. N_2O-ODHP performance of Fe/BN-IWI catalysts with varying Fe content, expressed in terms of reactants conversion and product selectivity, as a function of reaction temperature. Conditions: T = 673-873 K; m_{cat} = 0.2 g; F_T = 20 cm^3 min^{-1}; Feed = 8 vol% C_3H_8, 8 vol% N_2O, 84 vol% He; P = 1 bar.

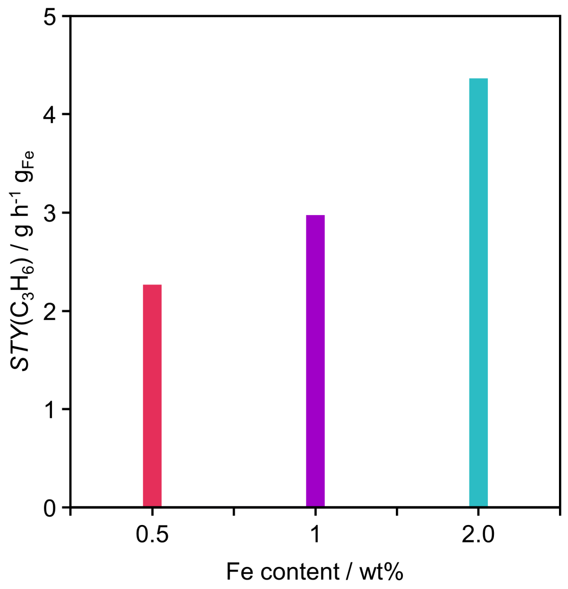

Figure S16. Space-time-yield (*STY*) of Fe/BN1 catalysts with varying Fe content, derived from the performance depicted in **Figure 4a**.

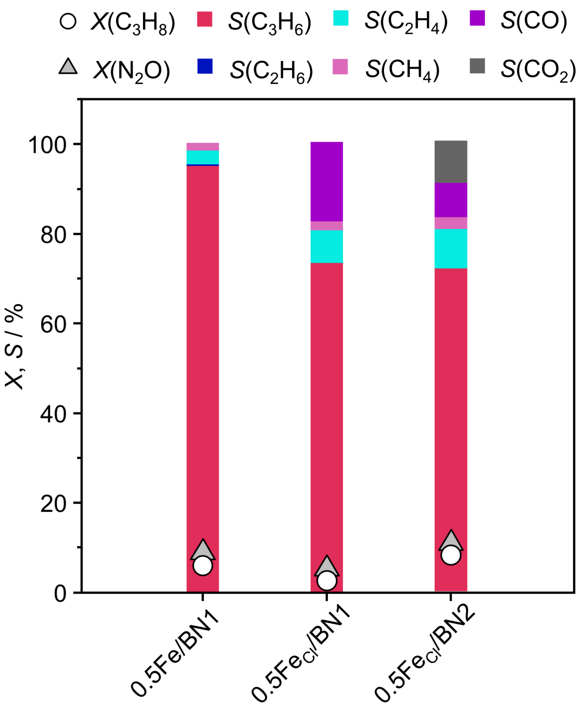

Figure S17. N_2O-ODHP performance of milled samples, synthesised with different metal precursors and milling times, represented by product selectivity patterns and C_3H_8 and N_2O conversion. Conditions: $T = 723$ K; $m_{cat} = 1$ g; $F_T = 20$ cm^3 min^{-1}; Feed = 8 vol% C_3H_8, 8 vol% N_2O, 84 vol% He; $P = 1$ bar.

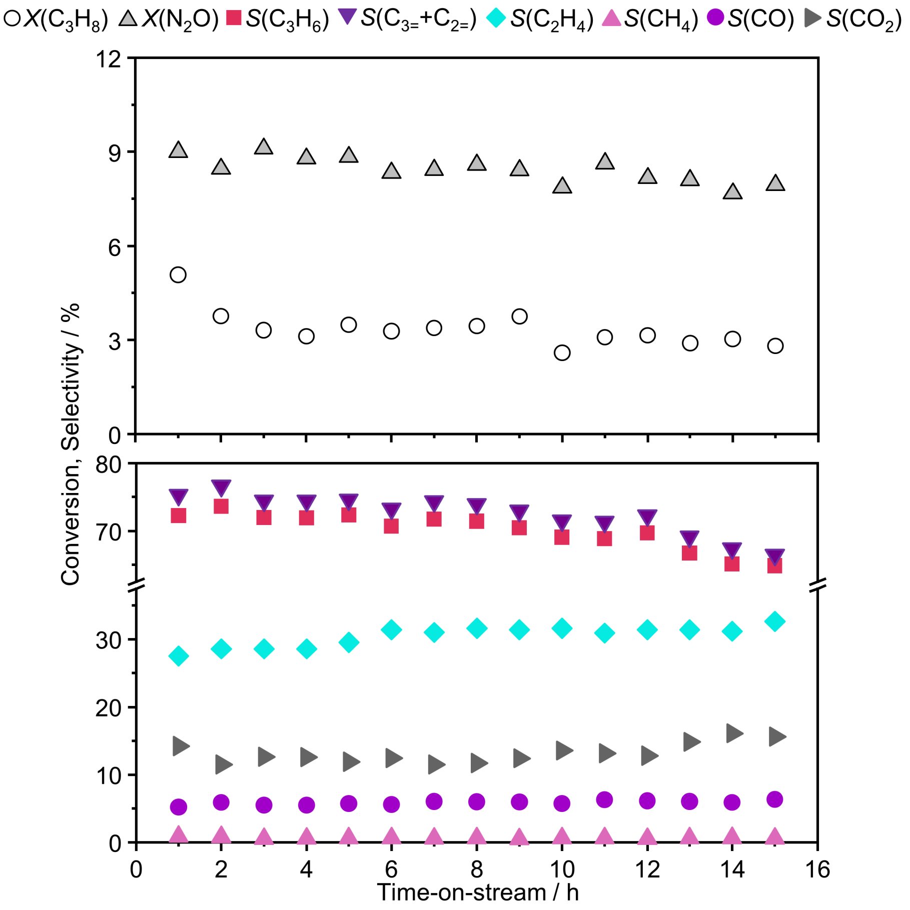

Figure S18. Reactants conversion and product selectivity during the stability test of 0.5Fe/BN-IWI in N_2O-ODHP. Products with selectivity below 1% are not reported. Conditions: $T = 723$ K; $m_{cat} = 1$ g; $F_T = 20$ cm^3 min^{-1}; Feed = 8 vol% C_3H_8, 8 vol% N_2O, 84 vol% He; $P = 1$ bar.

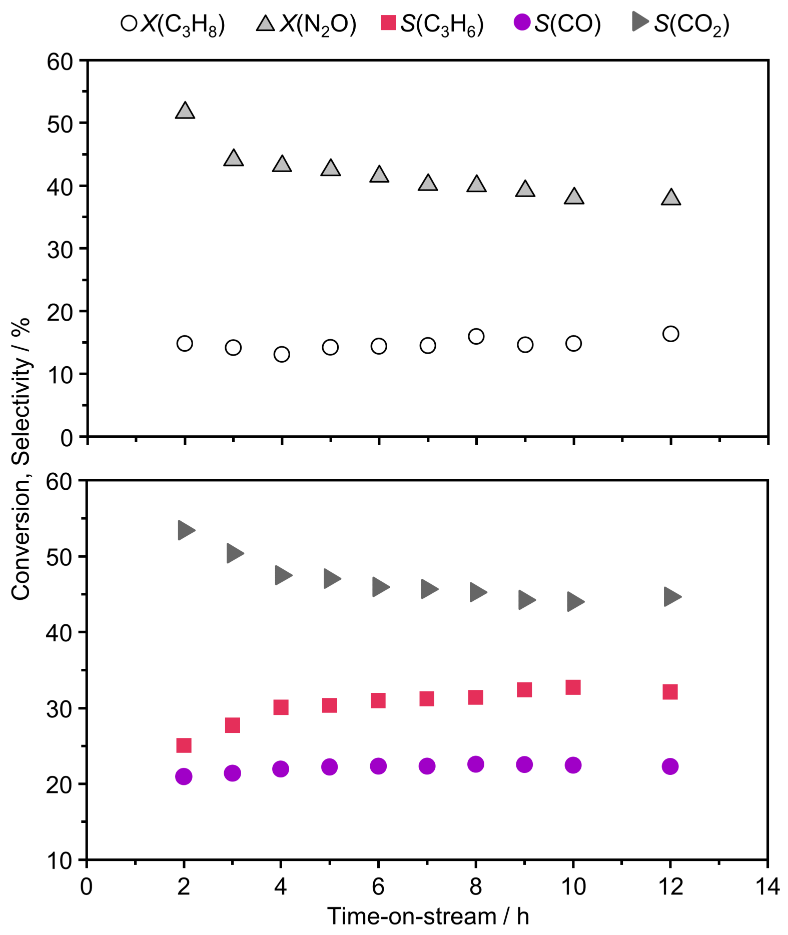

Figure S19. Reactants conversion and product selectivity during the stability test of 2Fe/BN1 in N_2O-ODHP. Products with selectivity below 1% are not reported. Conditions: $T = 723$ K; $m_{cat} = 1$ g; $F_T = 20$ cm^3 min^{-1}; Feed = 8 vol% C_3H_8, 8 vol% N_2O, 84 vol% He; $P = 1$ bar.

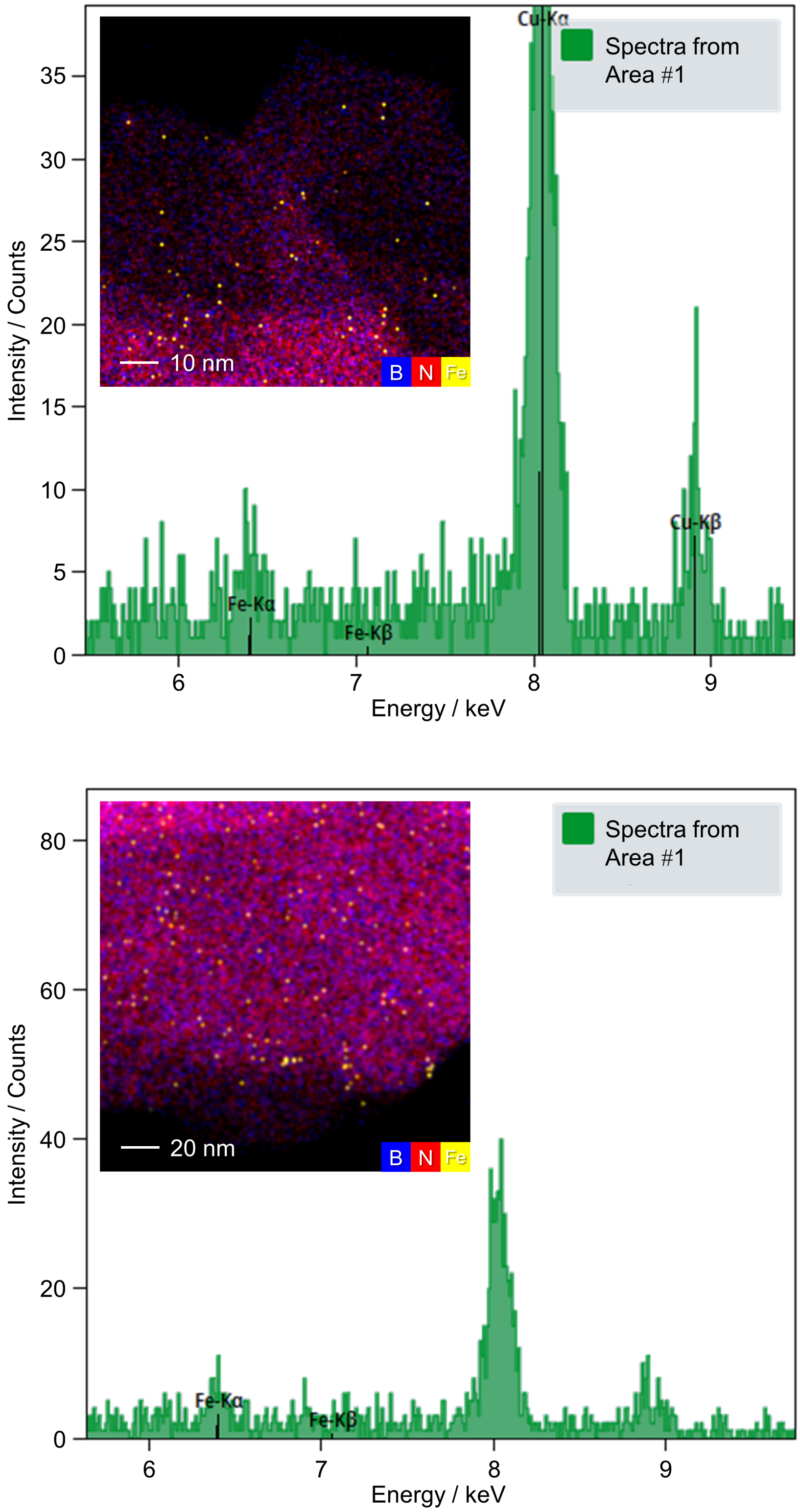

Figure S20. EDX maps and the relative integrated EDXS spectra of as-prepared (top), and used (bottom) 0.5Fe/BN1. Both spectra show a distinct signal for iron. Note that the speckles in the Fe signal should not be interpreted as individual atoms.

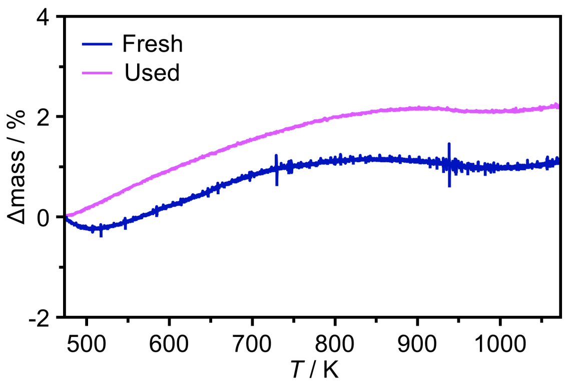

Figure S21. TGA of 0.5Fe/BN1 catalyst before and after use in N_2O-ODHP for 18 h. The slight increase in weight in the as-prepared and used samples suggests that complete oxidation of the catalyst surface occurred during the analysis.

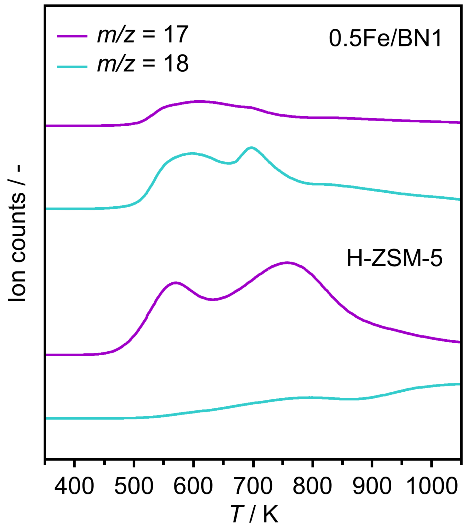

Figure S22. NH_3-TPD of selected as-prepared catalysts. Both m/z = 17 and 18 are shown, as OH^- and NH_3 may both contribute to the ion count at m/z = 17. The measurements reveal significant differences in acidity, with the m/z = 17 spectrum for H-ZSM-5 showing two clear distinct peaks: the first peak is attributed to Lewis and the second peak to Brønsted acidic sites.
